# A Functional Insulator Screen Identifies NURF and dREAM Components to Be Required for Enhancer-Blocking

**DOI:** 10.1371/journal.pone.0107765

**Published:** 2014-09-23

**Authors:** Dorte Bohla, Martin Herold, Imke Panzer, Melanie K. Buxa, Tamer Ali, Jeroen Demmers, Marcus Krüger, Maren Scharfe, Michael Jarek, Marek Bartkuhn, Rainer Renkawitz

**Affiliations:** 1 Institut für Genetik, Justus-Liebig-Universität, Giessen, Giessen, Germany; 2 Faculty of Science, Benha University, Benha, Egypt; 3 Proteomics Centre, Erasmus Medical Centre, Rotterdam, Netherlands; 4 Biomolecular Mass Spectrometry, Max Planck Institute for Heart and Lung Research, Bad Nauheim, Germany; 5 Helmholtz Centre for Infection Research, Braunschweig, Germany; The National Institute of Diabetes and Digestive and Kidney Diseases, United States of America

## Abstract

Chromatin insulators of higher eukaryotes functionally divide the genome into active and inactive domains. Furthermore, insulators regulate enhancer/promoter communication, which is evident from the *Drosophila* bithorax locus in which a multitude of regulatory elements control segment specific gene activity. Centrosomal protein 190 (CP190) is targeted to insulators by CTCF or other insulator DNA-binding factors. Chromatin analyses revealed that insulators are characterized by open and nucleosome depleted regions. Here, we wanted to identify chromatin modification and remodelling factors required for an enhancer blocking function. We used the well-studied *Fab-8* insulator of the bithorax locus to apply a genome-wide RNAi screen for factors that contribute to the enhancer blocking function of CTCF and CP190. Among 78 genes required for optimal *Fab-8* mediated enhancer blocking, all four components of the NURF complex as well as several subunits of the dREAM complex were most evident. Mass spectrometric analyses of CTCF or CP190 bound proteins as well as immune precipitation confirmed NURF and dREAM binding. Both co-localise with most CP190 binding sites in the genome and chromatin immune precipitation showed that CP190 recruits NURF and dREAM. Nucleosome occupancy and histone H3 binding analyses revealed that CP190 mediated NURF binding results in nucleosomal depletion at CP190 binding sites. Thus, we conclude that CP190 binding to CTCF or to other DNA binding insulator factors mediates recruitment of NURF and dREAM. Furthermore, the enhancer blocking function of insulators is associated with nucleosomal depletion and requires NURF and dREAM.

## Introduction

Chromatin insulators mediate boundary or barrier functions as well as an enhancer blocking activity [1]–[3]. Besides the highly conserved factor CTCF, the *Drosophila* genome also codes for the additional insulator factors zeste white5 (Zw5) and boundary element associated factor 32 (BEAF-32) [4]–[6]. Furthermore, suppressor of hairy wing [Su(Hw)] and the GAGA-binding factor (GAF) [7], [8] are responsible for the insulator activity at specific target sites. Binding sites for these factors are often found as “mixed” groups, but they are also found as single sites on their own [9]–[11]. A unifying cofactor seems to be centrosomal protein 190 (CP190), which co-localizes to many of the sites bound by the insulator factors [9]–[12]. Overall, there are about 6,000 CP190 sites in the *Drosophila* genome with 80% of them being bound by at least one of the five DNA-binding insulator factors. This suggests that CP190 may confer an important role in insulator function [13]. In fact, when comparing CTCF sites devoid of CP190 binding with those that are bound by both factors, nucleosomal occupancy at these sites is strikingly different [12]. CTCF plus CP190 binding correlates strongly with a nucleosome free region, whereas in the absence of CP190 the nucleosomal occupancy is indistinguishable between CTCF and non-CTCF sites [12]. Insulator function has often been correlated with the folding and looping of chromatin resulting in long-range chromatin interaction. A recent finding underscores this feature by showing that CTCF and CP190 are required to assemble repressed genes into Polycomb bodies [14]. In order to shed some light on the potential molecular mechanisms we wanted to identify co-factors for CTCF and/or CP190, which potentially might modify or remodel histones and nucleosomes.

We used an unbiased functional screen involving the CTCF/CP190-dependent insulator *Fab-8* and compared the identified set of factors with those detected by either purifying CTCF or CP190. With these three strategies we identified the nucleosome remodelling factor NURF and components of the multi-subunit transcription repressor complex dREAM. Chromatin “opening” and efficient insulator mediated enhancer-blocking are facilitated by these complexes.

## Results

### A genome-wide RNAi screen identifies many chromatin-associated factors required for enhancer-blocking

**Figure 1:**
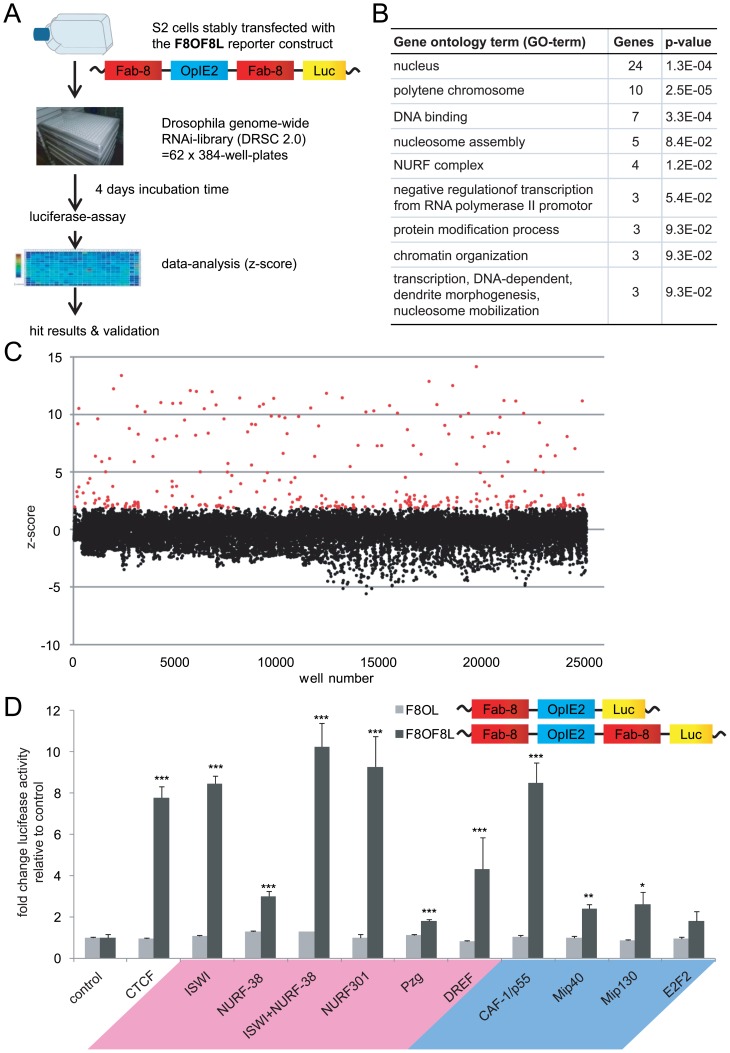
RNA interference (RNAi) identifies 78 factors inducing insulator reporter gene activity including NURF and dREAM components. (**A**) Workflow of the RNAi screen in 66×384-well plates from the DRSC. Knockdown of 13900 genes was done with *Drosophila* S2 cells with the integrated F8OF8L insulator reporter construct (F8, *Fab-8*; O, OpIE2 enhancer; L, *luciferase*). (**B**) Top GO-terms (determined via GeneCodis [58]–[60]) for the 78 identified genes. (**C**) High-throughput data shown in a dotplot diagram. Z-scores are indicated for every well (well number). For many gene products several wells contain different dsRNA sequences targeting the same gene. Z-scores higher than two are highlighted in red. (**D**) Individual depletion of NURF and dREAM components and associated factors verify enhancer blocking function. S2 cell pools with the integrated F8OF8L insulator reporter (dark grey) or the control F8OL reporter construct (light grey) were incubated with dsRNA against factors of the NURF-complex (pink): ISWI, NURF-38, CAF1/p55, NURF301, Pzg, DREF or against the dREAM-complex (blue): CAF1/p55, Mip40, Mip130, E2F2. Reporter gene activity is expressed as fold change relative to control knockdown. Error bars indicate the standard deviation of three individual replicates. (p-values: *≤0.05, **≤0.01, ***≤0.001).

In order to identify cofactors involved in CP190 mediated chromatin insulation we utilized a genomic region that has been well characterized for insulator function as well as for the functional dependency on CTCF and CP190. Such a region is the *Fab-8* insulator that separates the regulatory elements of the bithorax complex, which specifies the third thoracic segment and all eight abdominal segments of the fly [15], [16]. This element is bound by CTCF and CP190 [12], [17], and the enhancer blocking activity of *Fab-8* was shown to depend on CTCF and on CP190 [18]. Furthermore, the important role of CTCF for regulatory domain function in the bithorax complex is underscored by homeotic transformations of the abdominal segments in CTCF mutants, resulting in an additional abdominal segment 7 [18], [19]. This insulator element has been successfully used to analyse the enhancer blocking activity when inserted inbetween the OpIE2 enhancer and the SV40 promoter driven GFP reporter [20]. We modified this reporter and utilized the luciferase gene to facilitate easy measurements of insulator activity during the screening procedure. In addition to the enhancer blocking position of the *Fab-8* element placed between the enhancer and the reporter, a second *Fab-8* sequence upstream of the enhancer (F8OF8L) (Fig. 1A) allows for the use of an important control construct (F8OL), in which the *Fab-8* element is present at the upstream position but is missing at the enhancer blocking position. In this way enhancer blocking activity can be distinguished from general, unspecific repression effects or from enhancer or promoter repression, which should still occur with the F8OL construct. We used both constructs to generate stable clone pools with *Drosophila* S2 cells. The F8OF8L clone pool was tested for robustness of luciferase expression as well as for inducibility after CTCF depletion. In comparison, F8OL control clone pools were similarly tested and were found not to be inducible by CTCF depletion (see below and Figure S1). The genome-wide RNAi library DRSC 2.0, generated by the *Drosophila* RNAi Screening Center (www.flyrnai.org), was used. This library contains approximately 21,000 dsRNAs targeting 13,900 genes and is provided in a 384-well-plate format (Fig. 1A). After applying the F8OF8L clone pool to the plates for four days, luciferase activity was determined (Fig. 1C). The z-score shown is an indication of changes in luciferase activity compared to the mean in a group of scores. Most of the 21,000 dsRNAs resulted in a z-score range between +2 and −2, which was also seen without addition of dsRNA or with negative control dsRNAs directed against GFP or others. dsRNA directed against 78 of the genes expressed in S2 cells revealed an induction of the reporter gene with a z-score of two or higher (Table S1). These 78 genes can be grouped according to their GO-terms, which are highly enriched for chromatin modification, chromatin assembly and organisation, chromatin binding factors and regulation of transcription (Fig. 1B). Six of the top ranking 30 genes encoded components of two complexes, the nucleosome remodelling factor (NURF), and the multi-subunit transcription repressor complex *Drosophila melanogaster* RBF, E2F and MYB (dREAM). The NURF complex is composed of the chromatin remodelling ATPase ISWI and the components NURF-38, NURF-301 and CAF-1/p55 [21]; for review see [22]. This complex is well conserved and is the founding member of several ISWI containing remodelling complexes. The composition of the dREAM complex is multifaceted in containing *Drosophila* RBF, dE2F2, dMYB and the dMYB-interacting proteins MIP40, MIP120 and MIP130 [23], [24]; for review see [25]. We focussed on both of these complexes for the following analyses.

### Enhancer blocking function and protein interaction verify NURF and dREAM components as cofactors for CTCF and CP190

We verified the interference of enhancer blocking after RNAi treatment by individual knockdown experiments of NURF components ISWI, NURF-38, NURF301, CAF-1/p55 and dREAM components Mip40, MIP130 and E2F2 (Fig. 1D). As a positive control we depleted the cells from CTCF. We also included the Pzg and DREF factors, which are known to be associated either with NURF binding or function. The factor Pzg has been identified in the context of Notch signalling to be bound to the NURF complex [26] and DREF has been shown to interact with several components of NURF [27]. Furthermore, DREF has been found to localize to BEAF-32 binding sites, but with an anti-correlation in binding efficiency [28]. Here we found that dsRNA directed against DREF or, to a lesser extent Pzg, induced gene activity of the insulator reporter. Since the efficiency of depletion varies from factor to factor and between experiments, some of the functional effects were less strong. In line with this is the finding that the combined depletion of NURF-38 and ISWI resulted in an additional increase in luciferase activity (Fig. 1D). In order to distinguish whether NURF or dREAM have a general repressing effect on reporter, or whether the enhancer blocking function specifically requires both complexes, we tested the control F8OL cells devoid of the *Fab-8* element at the enhancer blocking position (Fig. 1D). Clearly, in contrast to the insulator reporter F8OF8L, the F8OL cells did not change the luciferase activity. For CTCF this confirmed our previous result that CTCF action is neither on the promoter nor on the enhancer function, rather, CTCF depletion impairs the enhancer-blocking function of the *Fab-8* element. Similarly, depletion from ISWI, NURF-38, CAF-1/p55, NURF-301, Pzg or DREF did not affect the expression activity of the F8OL control.

Thus, depletion of either NURF or dREAM factors, or the NURF associated factors Pzg and DREF, impaired the enhancer-blocking function of the *Fab-8* insulator.

**Figure 2:**
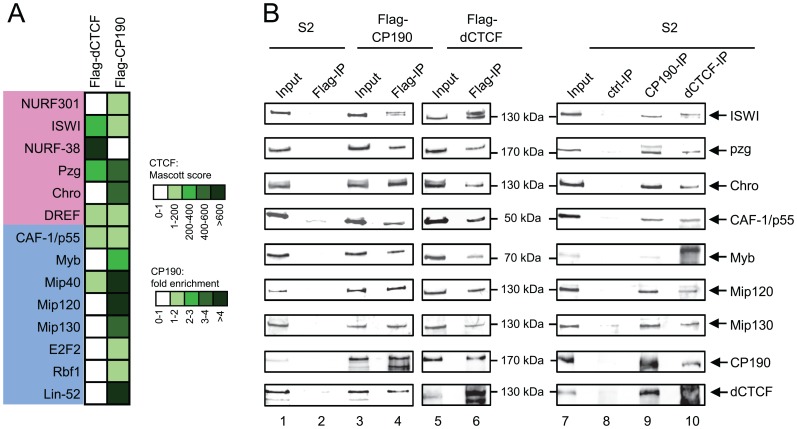
Purification of either CTCF or CP190 reveals NURF and dREAM binding to both insulator factors. (**A**) Interaction heatmap based on Mascot scores (dCTCF) or fold enrichment of normalized intensities (CP190), depicting associated factors identified by mass spectrometry after immunopurification of FLAG-dCTCF or FLAG-CP190 expressed in S2 cells. (**B**) Nuclear extracts from S2 cells (lanes 1–2, 7–10) and S2 cells stably expressing FLAG-CP190 (lanes 3–4) or FLAG-dCTCF (lanes 5–6) were precipitated with FLAG antibody (lanes 2, 4, 6), CP190 antibody (lane 9), dCTCF antibody (lane 10) or IgG (lane 8) as control. Antibodies used in Western blot are indicated on the right. Lanes 1, 3, 5 and 7: 1% Input.

dREAM or NURF, that are required for insulator function, might mediate their action by interacting with dCTCF or with CP190 that is bound to dCTCF. Alternatively, dCTCF or CP190, together with unknown factors, might prepare the epigenetic landscape such that dREAM or NURF target to specific chromatin modifications at insulator sites. In the first scenario it should be possible to purify dCTCF or CP190 together with associated dREAM and NURF factors, whereas in the second picture dREAM and NURF would not co-purify with dCTCF or CP190. To test this we generated Flag-tagged versions of dCTCF and of CP190 and expressed these separately in *Drosophila* S2 cells. After purification of each of the Flag-fusion proteins we used this material for mass spectrometric analysis and found dCTCF- and CP190-enriched factors. Among both, again, components of the dREAM and of the NURF complex were found. The NURF components were enriched by both dCTCF and CP190 purification (Fig. 2A). In contrast, dREAM components were primarily enriched with the CP190 purified material. There was one exception to this rule in the case of CAF-1/p55. This factor was found in dCTCF as well as in CP190 purified material. This is expected as this factor is found as a constituent of NURF as well as of dREAM [29], [30].

In order to verify the mass spectrometric analysis, we used the S2 cell clones expressing either Flag-CP190 or Flag-dCTCF for co-immunoprecipitation experiments. The Flag-precipitated material was analysed for the presence of NURF or dREAM components by Western blots. The availability and specificity of antibodies dictated the choice of components to be tested. In the case of NURF, we used antibodies against ISWI, CAF-1/p55 and the NURF associated factors Pzg and Chro [26], [31]. All of these resulted in a positive Western blot signal after Flag-immunoprecipitation of Flag-CP190 or of Flag-dCTCF. Co-precipitated ISWI frequently displayed a double band, potentially indicative of a modification. S2 cells without a reporter construct served as a negative control (Fig. 2B). Similarly, when testing for dREAM components Myb, Mip120 and Mip130, all resulted in positive Western blot signals with the Flag-CP190 or the Flag-CTCF material. This seems to contrast the mass spec results, which indicate a preferential enrichment after CP190 purification. An explanation for this may be that dREAM association is mediated by CP190 and that the more stringent purification of FLAG-dCTCF has lost these interactors, whereas the direct co-precipitation allowed for CTCF mediated precipitation of CP190 bound DREAM factors. For further verification we also checked for co-precipitating proteins of the endogenous CP190 or dCTCF factors. Except for weak signals detected with the Myb antibody, all other antibodies confirmed specific Western blot signals after precipitation of CP190 or of dCTCF. Control precipitations with mouse IgG remained negative (Fig. 2B).

Thus, the unbiased dsRNA screen, the functional enhancer-blocking verification, the mass spectrometry results, the co-IP experiments of Flag-tagged proteins and of the endogenous CP190 and dCTCF proteins identified NURF and dREAM components to be functionally associated with CP190 and dCTCF in the context of enhancer blocking.

### NURF and dREAM components co-localize with CP190

**Figure 3:**
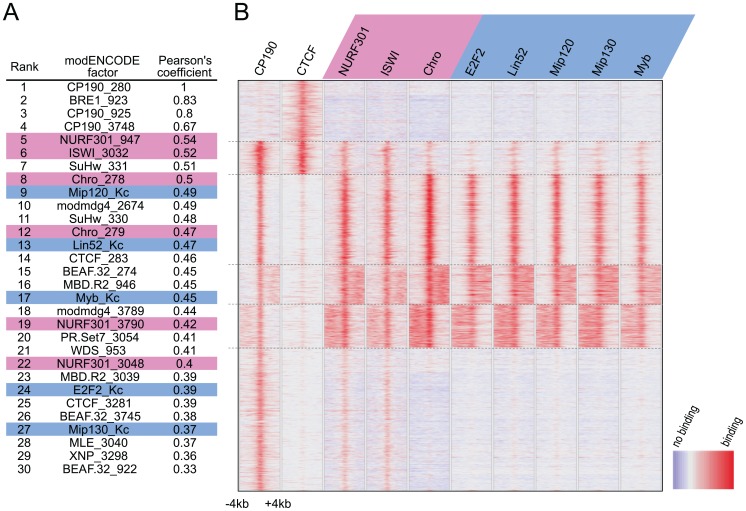
NURF and dREAM components co-localize with CP190 genome-wide. (**A**) Correlation analysis for genome-wide binding of CP190 with 215 profiles from S2 cells (modENCODE) and 5 profiles from Kc cells [33]. Shown are the top 30 ranking factors. Components of the NURF complex are marked in pink and of the dREAM complex in blue. (**B**) Cluster heat map of 6,000 genomic regions with CP190 and/or CTCF sites compared with binding sites for components of NURF (NURF301, ISWI, Chro) and dREAM (E2F2, Lin-52, Mip120, Mip130, Myb) complexes. Each lane represents an 8 kb region. Scale represents binding (red) to no binding (blue).

The functional effects after depletion of NURF or dREAM components as described above, as well as the co-purification with CTCF or CP190, suggested that these factors should at least in part co-localize at binding sites in the genome. Therefore we took advantage of the modENCODE project data [32] and compared CTCF/CP190 binding data to more than 200 other ChIP-chip profiles published for S2 cells within the projects database. In addition we included the available dREAM binding data derived from ChIP-chip experiments in Kc167 cells [33]. We calculated the correlation coefficients between the CP190 and all of the other binding profiles after calculating the mean binding within 100 bp bins (Figure S2). When we ranked the factors for the calculated coefficients we found alternative CP190 ChIP-chip profiles or previously known CP190 interaction partners amongst the highest scoring factors (Fig. 3A), such as the insulator factors Su(Hw), Mod(mdg4), CTCF and BEAF-32. Interestingly, NURF301, ISWI, the NURF interacting factor Chro and the dREAM profiles Mip120, Mip130, Lin-52, Myb and E2F2 were similarly or even better correlated as compared to the other insulator factors.

Additionally we were interested to see the binding distribution of NURF and dREAM-components in the context of CP190 binding sites. Therefore, we compiled the binding data for these factors within an 8 kb window around at the about 6000 CTCF/CP190 peaks. We performed cluster analysis using k-means and found a separation into 6 clusters to yield the most informative view of the data (Fig. 3B). Cluster 1 is marked by strong CTCF binding but majorly devoid of any binding for CP190 or dREAM/NURF-related cofactors. In contrast, cluster 2 is bound by CTCF and CP190 as well as by all dREAM and NURF components tested for genome-wide binding. Clusters 3, 4 and 5 show a weak binding of CTCF, but nevertheless a strong binding for all dREAM and NURF components, suggesting that CP190 is the determinant for dREAM and NURF co-localization. Interestingly, clusters 4 and 5 show a second CP190 binding site within the 8 kb window, which in each case is marked by NURF and dREAM. This second site is found either on the “right” (cluster 4) or on the “left” (cluster 5) at a variable position relative to the first CP190 site. Cluster 6 has only CP190 with weak binding of NURF components, but no dREAM binding. All together, the large majority of CP190 sites are marked by NURF binding, a smaller but still large fraction is marked by additional binding of dREAM-components. To further analyze the colocalization of CTCF, CP190, NURF and DREAM we carried out a correlation analysis of several NURF and DREAM factors at CP190 only sites, at CTCF only sites and at CTCF/CP190 double sites (Figure S3). A striking correlation between CP190 and NURF and DREAM factors is evident. In contrast, there is no correlation or anti-correlation detected with CTCF. That suggests that the majority of the insulator factors recruiting CP190 are targeted by NURF and DREAM as well. Furthermore we analyzed the relationship between CP190 binding and the co-occurrence of NURF with respect to functional annotation of associated genomic regions (Figure S4). CP190 sites are enriched at transcriptional start and upstream sites in the range of +1 kb to −10 kb. This enrichment is similarly seen for Nurf301, again suggesting that CP190 sites are NURF sites as well.

Together these data suggest that chromatin binding patterns of CP190, NURF and dREAM components are highly similar and therefore are suggesting a common regulatory function.

**Figure 4:**
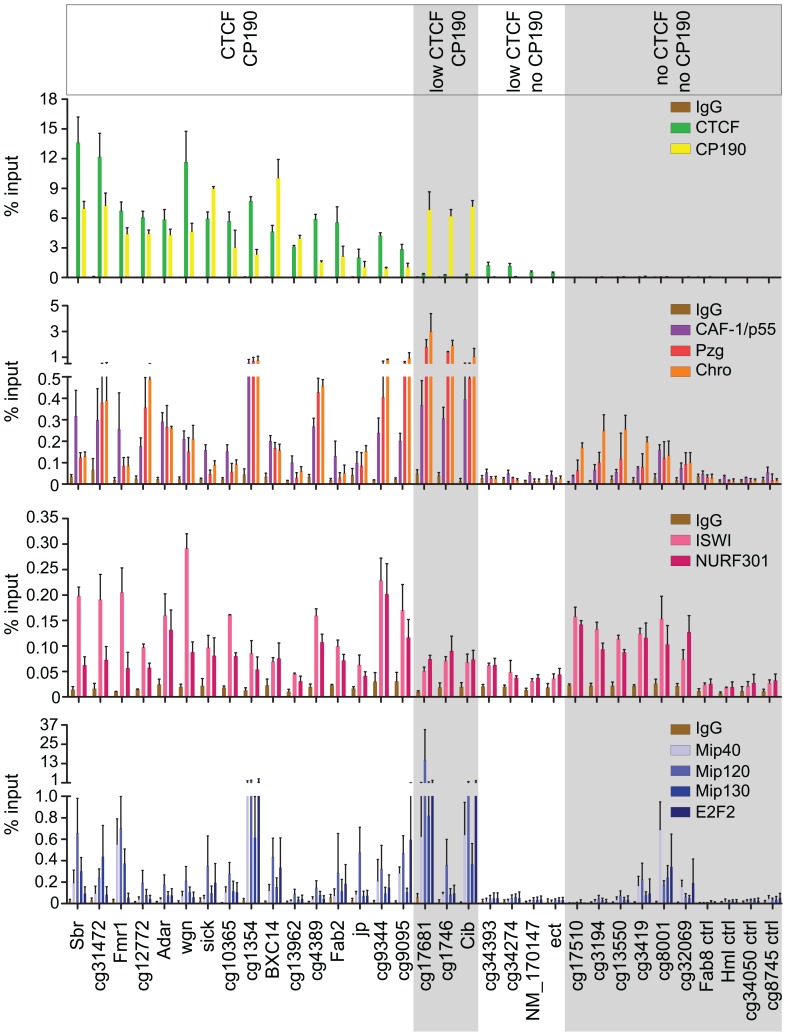
NURF and dREAM components co-localize with dCTCF/CP190. ChIP in S2 cells with antibodies against CTCF and CP190 and components of the NURF complex (ISWI, NURF301, Pzg and Chro) and dREAM complex (Mip40, Mip120, Mip130, E2F2) or CAF-1/p55. The genomic regions tested are indicated (compare Table S2) and grouped into CTCF plus CP190, low CTCF plus CP190, low CTCF without CP190 and neither CTCF nor CP190. Error bars indicate the standard deviation of three independent experiments.

In order to verify co-localization of NURF and dREAM with CP190 and dCTCF we precipitated chromatin with various antibodies. We tested the precipitate for the presence of sequences predicted from the databases to be bound by dCTCF and CP190 (sequences are listed in Table S2). The NURF associated factors Pzg and Chro were significantly enriched at many CP190 sites (Fig. 4). Control sites (no CTCF, no CP190) were chosen which are not bound by either dCTCF or CP190, but bound by either NURF or DREAM or bound by none of these factors. Within this control group Pzg and Chro were specifically bound to NURF sites. Precipitation of the NURF factors ISWI, NURF301 and CAF-1/p55 revealed binding to most CP190 bound sites as was the case for precipitation of the dREAM factors Mip40, Mip120, Mip130 and E2F2.

**Figure 5:**
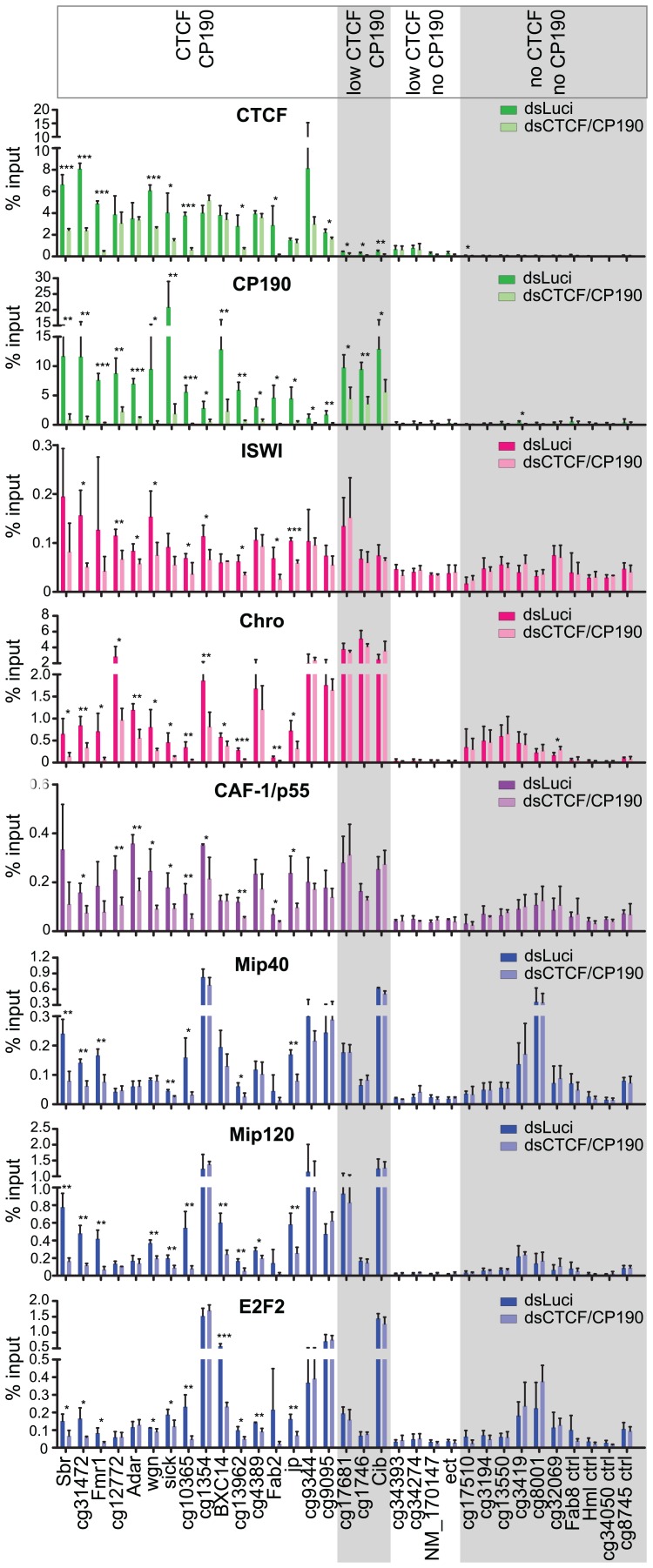
Recruitment of NURF and dREAM is dependent on dCTCF and CP190 at specific sites. ChIP in S2 cells treated with dsRNA against dCTCF and CP190 (dsCTCF/dsCP190; dark colors) or against luciferase as control (dsLuci; light colors). Antibodies were used specific for dCTCF, CP190 and components of the NURF (ISWI, Chro) and dREAM complex (Mip40, Mip120, Mip130) or, as part of both complexes, CAF-1/p55. Error bars indicate the standard deviation of three independent experiments. (p-values: *≤0.05, **≤0.01, ***≤0.001; ND: not determined).

Given the frequent colocalization of NURF and dREAM with dCTCF and CP190, and given that these factors co-purify, we predicted that NURF and dREAM are targeted to chromatin by binding to CP190 and/or dCTCF. Consequently, when depleting cells of dCTCF and CP190, recruitment of NURF and dREAM to these sites should be impaired, unless other factors contribute to binding of NURF and dREAM to insulator chromatin. We tested the sites characterized above for NURF and dREAM binding after double knockdown of dCTCF and of CP190 (Fig. 5; Fig. S5). Most of the double bound CTCF/CP190 sites showed a significant reduction of NURF and dREAM components ISWI, Chro, CAF-1/p55, Mip40, Mip120 and Mip130. CP190 only sites did not change binding of the NURF and dREAM factors. This might be due to the fact that CP190 depletion on these sites is quite inefficient, only reducing bound CP190 to about 50% (Fig. 5), in contrast to the double CTCF/CP190 sites, which upon additional depletion of CTCF reduce CP190 binding below 30%. Most importantly, NURF and dREAM only sites (no CTCF and no CP190) do not show a significant change in NURF and dREAM binding, suggesting that CTCF or CP190 are the recruiting targets for NURF and dREAM at these sites. As an additional control we tested for a potential effect of depleted NURF and dREAM factors on CTCF or CP190 binding. The results showed that depletion of ISWI (NURF) or of MIP130 (DREAM) did not affect CTCF or CP190 binding (Figure S6).

Thus, binding of NURF or dREAM components at dCTCF/CP190 binding sites was shown to be dependent on the presence of dCTCF or CP190.

### The effects of NURF or dREAM on insulation are site specific

**Figure 6:**
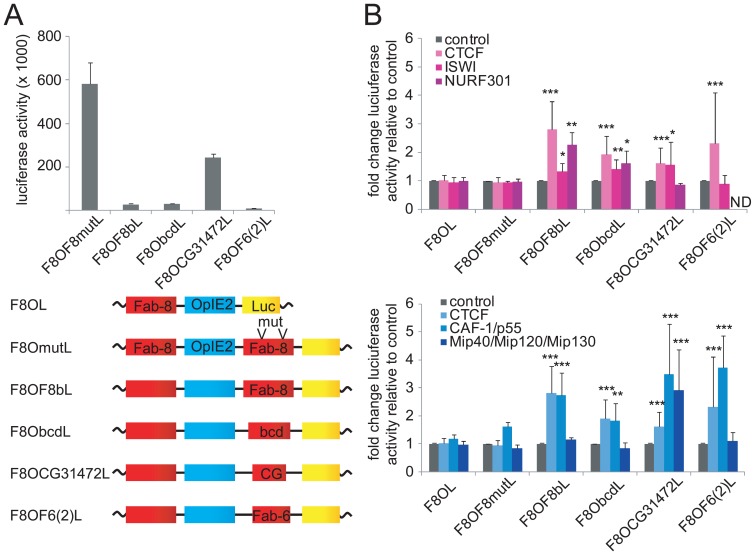
Insulator specific effects of NURF and dREAM components. S2 cells pools with the integrated luciferase reporter constructs with different CTCF/CP190 binding sites located between the enhancer (O, OpIE2) and the promoter of the reporter gene (L, *luciferase*). (**A**) Luciferase activity after control knockdown of GFP shows the enhancer blocking activity of *Fab-8* (F8), bicoid (bcd), CG31472 and *Fab-6* (F6(2)), when compared to the CTCF binding site mutant (F8mut) (top). Error bars indicate the standard deviation of three biological replicates. The different insulator reporter constructs are depicted (bottom), the genomic fragments used are indicated in Table S2 and genome browser views are in Figure S7. (**B**) Knockdown experiments against CTCF, ISWI or NURF301 (top) and of CTCF, CAF1/p55 or triple-knockdown of Mip-factors (bottom). Fold change of luciferase activity is calculated relative to the control knockdown. Error bars indicate the standard error of three or more individual replicates (p-values: *≤0.05, **≤0.01, ***≤0.001; ND: not determined).

As determined above, a subset of dCTCF/CP190 bound sites recruit NURF or dREAM such that the *Fab-8* insulator requires NURF and dREAM for enhancer-blocking. Since there are site-specific differences in NURF and dREAM recruitment, we wanted to test the functional consequences of NURF or dREAM depletion on enhancer-blocking at different insulator sites. In order to use an identical vector backbone with an exchangeable insulator cassette, we modified a construct with mutated *Fab-8* CTCF binding sites [20]. After the exchange of the reporter-gene the F8OF8mut cassette could be replaced by other sequences and, as a control, by the *Fab-8* insulator now labelled F8OF8bL. This showed a strong enhancer blocking activity when compared to the mutant F8OF8mutL (Fig. 6A). For the experiments with other potential insulators we selected strong CTCF binding sites, which we had previously observed [12]. The site F6(2) is from the bithorax locus and corresponds to the *Fab-6* element known to be involved in segment specific chromatin insulation, which has been tested for enhancer blocking activity [34]-[36]. Here we show that *Fab-6* in our assay has an insulator activity as well (Fig. 6A). The same is true for the CTCF sites at the promoter region of bicoid (bcd) and upstream of the gene CG31472. Both sites were not described as insulators before and are within the sequence cluster 2 as identified in Fig. 3B. Furthermore, CG31472 was one of the strongest deregulated genes after depletion of CTCF and Cp190, respectively [12]. Genome browser views concerning known insulator factors reveal CP190 binding in all cases (Figure S7). This set of constructs was challenged by depletion of CTCF, ISWI, Nurf-301, CAF-1/p55 and the three Mip factors of the dREAM complex, Mip40, Mip120 and Mip130. All of the constructs showed an increase in gene activity upon CTCF depletion (Fig. 6B). Depletion of Nurf-301 impaired the *Fab-8* and bcd insulators, ISWI impaired *Fab-8*, bcd and CG31472, CAF-1/p55 effected *Fab-8*, bcd, CG31472 and *Fab-6*. Triple depletion of the Mip factors only showed an effect on the CG31472 element.

Therefore, different combinations of NURF and of dREAM components contribute to site-specific enhancer-blocking activity.

### Depletion of CTCF/CP190 causes changes in nucleosomal occupancy similar to depletion of ISWI

**Figure 7:**
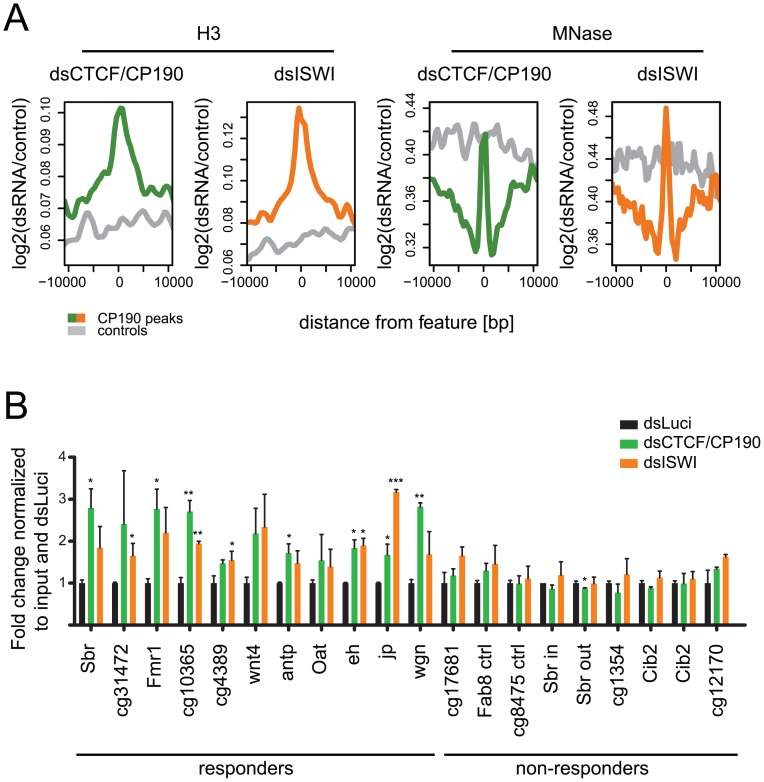
NURF binding causes nucleosomal depletion at CP190 binding sites. (**A**) Cumulative representation of changes in H3-binding and MNase-protection as detected by H3 ChIP-seq and MNase-seq after depletion of CTCF/CP190 (green) or ISWI (orange). Data is shown as coverage for specific knock-down normalized to luciferase control knock-down (luci) after log2-transformation. Average effects are shown across CP190 binding sites (colored) or control sites shifted 25 kb (grey). (**B**) All sites with increased H3 binding after CTCF/CP190 depletion (responders) show a similar H3 increase upon ISWI depletion. Non-responding sites after CTCF/CP190 depletion (non-responders) do not respond to ISWI depletion. H3 ChIP in S2 cells treated with dsRNA against CTCF and CP190 (dsCTCF/CP190; green), ISWI (dsISWI; orange) or against luciferase as control (dsLuci; black). Error bars indicate the standard deviation of two independent experiments (p-values: *≤0.05, **≤0.01, ***≤0.001; ND: not determined).

Previously, we were able to identify a molecular function of CP190. We could show that CTCF sites bound by CP190 caused this region to be depleted of nucleosomes, whereas CTCF sites devoid of CP190 show a regular nucleosomal pattern [12]. Here we found that the NURF complex with the nucleosomal remodeling ATPase ISWI is found at CP190 sites and that it is required for enhancer blocking activity of the *Fab-8* insulator. In order to understand whether ISWI targeting to CP190 sites may cause the CP190 specific nucleosomal depletion we studied the consequences of reducing ISWI amounts from *Drosophila* S2 (Fig. S8). As a read-out we analyzed the genome-wide distribution of and occupancy by nucleosomes. We performed both a histone H3 specific ChIP-seq and the analysis of DNA sequences covered by mono-nucleosomes after digestion with micrococcal nuclease (MNase). When we compiled the H3-binding profile across all CP190 binding sites we detected a significant increase of H3-binding when comparing the CTCF/CP190 RNAi treated sample with the luciferase RNAi control, with the maximum increase occurring as expected at the site of CP190 binding (Fig. 7A). Strikingly, a very similar effect can be observed after depletion of ISWI at the CP190 sites. To test whether the observed H3 increase is a measure for nucleosomal occupancy at these sites, we compared the increase of MNase resistant DNA after CTCF/CP190 depletion with the ISWI depletion. Again, a site specific increase in local MNase-protection for both CTCF/CP190- as well as ISWI-specific RNAi centered over CP190 binding sites became evident. Corresponding control sites did not show such an increase (Fig. 7A). In order to exclude unspecific effects that might be attributed to any open chromatin in general, we analyzed open chromatin sites as mapped by DNase I hypersensitivity [37] and compared these sites overlapping with CP190 binding with non-overlapping sites. Again, the increase in H3 binding or in MNase resistant sequence reads after depletion of CTCF/CP190 or of ISWI was specifically enriched at CP190 sites (Figure S9), although a general effect was seen as well.

To verify these observations at individual CP190 binding sites we analyzed H3-ChIP material at 20 genomic loci. We again compared CTCF/CP190 depletion with ISWI depletion. We grouped the loci according to the increase in H3-ChIP after CTCF/CP190 depletion into responding sites and non-responding sites (Fig. 7B). When inspecting the H3 ChIP efficiency after ISWI depletion all of the positive sites showed an H3 increase after ISWI depletion, whereas all negative sites did not respond to the depletion of ISWI. We also did a side-by-side analysis of H3 ChIP and of MNase digestion (Figure S10) showing that both procedures complement each other in showing that CTCF/CP190 depletion and ISWI depletion result in a similar increase of nucleosomal occupancy. When analyzing these changes in respect to the 6 binding site clusters defined in Fig. 3 we find the expected result: The clusters two to five with strong binding of CP190 and of NURF show a significant increase of H3 binding upon CTCF/CP190 depletion (Figure S11A), similar to the ISWI depletion (Figure S11B). The broad and skewed peaks in clusters four and five reflect the CP190 and NURF binding pattern in these clusters.

Thus, we can conclude that the CP190 mediated depletion of nucleosomes at CP190 binding sites is mediated, at least in parts, by ISWI, the ATPase of the NURF complex.

## Discussion

In order to identify factors required for the function of chromatin insulators we used three different unbiased screening procedures. The RNAi screen, the mass spectrometric analysis of proteins associated with CTCF or CP190, and the genome-wide bioinformatics analysis of factors colocalizing with CTCF or CP190. All of these identified components of the multi-subunit complexes NURF and dREAM. Both are known to mediate chromatin modification and transcriptional regulation. For insulator elements it has been shown that distinct chromatin signatures are found [32], although most of these specific marks can be attributed to the location of such elements either in the vicinity of transcriptional start sites (TSS) or at further upstream positions. Thus, it cannot be predicted [32] which type of chromatin modification might be generated by a CP190 associated chromatin modification complex.

The dREAM complex of *Drosophila* has been characterized to be built up from *Drosophila* RBF, dE2F2, and dMyb-interacting proteins. The first identification was in the context of transcriptional repression [23], [24], with MIP130 antibody staining of transcriptionally silent sites on polytene chromosomes. Target gene analysis revealed that dREAM is required to repress differentiation specific genes, whereas gene expression profiles in addition to gene repression also revealed an activation function of dREAM [33]. Targeting of dREAM to chromatin has been shown to be site specifically mediated by either E2F2 or by Myb [33]. The Myb mediated function and targeting of dREAM did not require the DNA binding domain of Myb, suggesting other targeting mechanisms [38]. Different repression mechanisms were suggested from the analysis of cell-cycle regulated and from cell-cycle independent genes [39], [40]. Here we show that at least a fraction of dREAM complexes is recruited by CP190 and mediates some of the insulator/enhancer blocking activity. E2F, pRB and dREAM activity has been reported to be mechanistically associated with histone modification [39], [41]–[43], however it is not known how this complex affects chromatin structure (for review see [25]). It can be envisaged that the dREAM complex contributes to the chromatin modification state at CP190 binding sites.

Besides specific chromatin modification, insulator sites in the genome are frequently characterized by depletion of nucleosomes, irrespective of the insulator position close to the TSS or at further upstream positions [32]. The second complex identified with our functional and binding screening was the NURF complex. This complex is the founding member of several ISWI containing remodelling complexes. The ATPase activity of ISWI is known to move or eject nucleosomes. Thus, ISWI containing NURF was potentially a good candidate to mediate the nucleosomal depletion at CP190 binding sites. Previously, *Drosophila* ISWI has been shown in a functional reporter assay to interfere with enhancer blocking activity of the *Fab-8* insulator [44]. Here we used the *Fab-8* insulator for an unbiased screen and identified all four NURF components to be required for enhancer blocking activity.

The NURF complex is composed of the chromatin remodelling ATPase ISWI and the components NURF-38, NURF-301 and CAF1/p55 [21]; for review see [22]. For CP190 bound dCTCF sites we could previously show that the depletion of nucleosomes at these sites is CP190 dependent [12]. Either the analysis of CTCF sites devoid of CP190 or after CP190 depletion revealed nucleosomal occupancy or an increase in the amount of histone H3 [12]. Here we could show that NURF binding to CP190 sites requires CP190. Genomic regions responding to CTCF/CP190 depletion with an increase in H3 or in MNase resistant DNA show a similar response to ISWI depletion. This raises the question of the functional impact of a nucleosome depleted region in the context of chromatin insulation or enhancer blocking. dCTCF binding to chromatin is not affected by a nucleosomal increase upon CP190 depletion [12]. Chromatin insulation at boundary positions can be envisaged to require active nucleosomal depletion in order to prevent the spreading of repressive chromatin modification such as H3K27me3 from an inactive chromatin domain through the insulator into an active domain [12]. For the enhancer blocking function of an insulator it is well established that long distance interaction and chromatin looping are required [2], [45]–[49]. How this activity might be connected to nucleosomal depletion at the insulator site can only be speculated. It has been proposed that insulators may have evolved from specialised derivatives of promoters [50]. In general, promoters are depleted from nucleosomes (for review see [51]), thereby allowing for efficient binding of a multitude of promoter factors. The concentration of these factors is dramatically increased by the clustering of active promoters in nuclear space [52]. Similarly, one might speculate that nucleosomal depletion by insulator bound CP190 and nuclear clustering [48] may fuel the eficiency of additional insulator factors to bind to their target sites.

Based on these and on published observations we conclude that CP190 binds to CTCF or to other DNA binding insulator factors serving as a binding platform for compexes with enzymatic function, like NURF and dREAM. This recruitment causes chromatin modification, such as nucleosomal depletion observed at binding sites for insulator factors.

## Materials and Methods

For additional details of methods and primers see: Text S1: Supplementary information on methods and references.

### DNA Plasmids

F8OL, F8OF8L and F8OF8mutL were generated by replacing GFP from F8enhGFP, F8enhF8GFP and F8enhF8mutGFP [20] with luciferase from pGL3 (Promega) after digestion with XbaI and HindIII. This reporter determines the interference of the enhancer/promoter interaction between the OpIE2 enhancer, which is commonly employed in insect expression vectors, and the SV40 minimal promoter. F8OF8bL, F8ObcdL, F8OF6(2)L and F8OCG31472L were generated by replacing the mutated *Fab-8* sequence from F8OF8mutL with CTCF binding site fragments *Fab-8*, bicoid, *Fab-6* and CG31472 after digestion with SalI and BglII (see Table S2 for primer sequences).

### Cell culture

*Drosophila* S2 cells were transfected with the DNA plasmids using the CaPO_4_ method and selected with puromycin. S2 clone pools were raised and cultured in Schneider's Medium (Invitrogen; supplemented with 10% fetal bovine serum (FBS), 1% penicillin/streptomycin and glutamine). Synthesis of dsRNA and RNAi treatment was done as described on www.flyrnai.org (see supplements for primer sequences).

### RNAi screening

The genome-wide dsRNA library (DRSC 2.0) was produced by the Drosophila RNAi Screening Center (DRSC) at Harvard Medical School (www.flyrnai.org). The library comprises 66×384-well plates and covers the entire genome. A detailed description of the RNAi screening, RNAi hit validation and RNAi on other insulation sites can be found in the supplementary methods (Text S1). A basic workflow is shown in figure 1A.

### ChIP and MNase assay

S2 cells were cultured and processed for chromatin immunprecipitation as described previously [12] (see supplements). For MNase digestion S2 cells were fixed with 0.3% formaldehyde. After preparation of nuclei the DNA was digested with MNase. Proteins and RNA were degraded, the resulting DNA purified and electrophoresed on an agarose gel. Mononucleosome bands were excised from gel and processed for sequencing (see supplements).

### Co-IP and Mass Spectrometry

S2 cells were stably transfected with pRm-HA/FLAG-dCTCF or -CP190 and the expression of cell clones induced with 500 mM CuSO_4_ for 24 h. For endogenous Co-immunoprecipitation wildtype S2 cells were used. Nuclear extract preparation was performed as described [53]. The nuclear lysate was incubated together with Protein G Plus/Protein A Agarose-coupled FLAG-M2 antibody (Sigma) or CP190 and dCTCF specific antibodies. After overnight incubation several washing steps were performed (20 mM Tris-HCl (pH 7.5), 100 mM NaCl, 0.25% NP-40). Proteins were precipitated with trichloroacetic acid (TCA). The precipitated proteins were separated on SDS-PAGE gels and analyzed either by Western-blotting or by mass spectrometry (see supplements).

### Antibodies

Rabbit anti-dCTCF [18], mouse anti-CP190 [54], guinea pig anti-NURF301 [55], rabbit anti-ISWI [55], rabbit anti-Pzg and rabbit anti-Chro [56], rabbit anti-CAF-1/p55 [57], rabbit anti-Myb, anti-Mip40, anti-Mip120, anti-Mip130 [29] and rabbit anti-H3 (Abcam, ab1791) were used.

### Data deposition

Data has been deposited in NCBI's Gene Expression Omnibus (GEO) database under accession number GSE51600: http://www.ncbi.nlm.nih.gov/geo/query/acc.cgi?acc=GSE51600.

## Supporting Information

Figure S1 **F8OL and F8OF8L clone pools show the expected CTCF dependent enhancer blocking.** S2 cell clone pools with the integrated F8OF8L insulator reporter or the control F8OL reporter construct were incubated with dsRNA against GFP (control) or against CTCF (CTCF). Reporter gene activity is expressed as relative light units. Error bars indicate the standard deviation of three individual replicates.
(TIF)

Figure S2 **CP190 binding profiles are very similar to DREAM and ISWI/Nurf301 profiles.** Publicly available ChIP-chip data for DREAM components (Mip130, Mip120, E2F2, Myb, Lin-52) (Georlette et al. 2007) as well as CP190, CTCF, ISWI, Nurf301 and several other profiles (ModEncode) serving as controls were binned into 100 bp bins by calculating the average enrichment of ChIP over input within each bin. Pair wise correlation coefficients were calculated. Hierarchical clustering of coefficients is shown as color coded heat map. The numbers behind ModEncode derived profile names refer to ModEncode IDs.
(TIF)

Figure S3 **DREAM and NURF associate with CP190 and CTCF/CP190 but not with stand-alone CTCF binding sites.** Cumulative binding profiles for indicated factors across the 3 classes of CTCF/CP190 binding sites (CTCF only, CP190 only and common CTCF/CP190). Stand-alone CP190 as well as common CTCF/CP190 sites are bound by DREAM and NURF components to a similar extent whereas stand-alone CTCF sites are devoid of both complexes. All binding data are from ModENCODE.
(TIF)

Figure S4 **CTCF as well as CP190 sites bound simultaneously by NURF are enriched for promotor associated annotations.** Distribution of genomic elements (red for intergenic, yellow for transcriptional start site (TSS) upstream region (−1 kb to −10 kb upstream of TSS), green for TSS (+/−1 kb around TSS), light blue for exon and dark blue for intron and purple for transcriptional end sites (TES)) across CTCF and CP190 binding sites with respect to overlap with NURF301 binding (data from ModENCODE). Enrichment for TSS-associated binding of CTCF and CP190 is associated with simultaneous NURF301 binding.
(TIF)

Figure S5 **Western blot after knockdown of CTCF and CP190 demonstrates CP190 and CTCF depletion.** S2 cells were transfected with dsRNA corresponding to dCTCF and CP190 (dsCTCF/CP190) or firefly luciferase (dsLuci) as control. Cell extracts of three independent experiments were analyzed by Western blot with antibodies directed against dCTCF, CP190 or tubulin as loading control.
(TIF)

Figure S6 **Depletion of ISWI or MIP130 does not affect CTCF or CP190 binding.** (A) Western blot after knockdown of ISWI (dsISWI; NURF complex) and Mip130 (dsMip130; dREAM complex) demonstrates ISWI and Mip130 depletion, but no influence on CTCF/CP190 protein level. knockdown control, dsLuci; protein loading control, Tubulin. (B) ChIP in S2 cells treated with dsRNA against ISWI (dsISWI) and Mip130 (dsMip130) or against luciferase as control (dsLuci). Antibodies were used specific for dCTCF (top) and CP190 (bottom). The genomic regions tested are strong binding sites for dCTCF and CP190: Sbr, cg31472, Adar, cg12772, wgn, CG1354; a weak binding site for dCTCF: cg17681 and two negative control sites: Fab-8 ctrl and cg8745 ctrl. Values are expressed as % input. Error bars indicate the standard deviation of three independent experiments.
(TIF)

Figure S7 **Genome browser view of insulators Fab-8, bcd, CG31472 and Fab-6.** Publicly available ChIP-chip data for CP190, CTCF and other insulator binding proteins (BEAF, Zw5, Su(Hw), Modmdg4 and GAF) (ModEncode) show the binding profiles at the tested insulator elements (bottom black box in each case). Known transcripts are shown at the top in each case. (**A**) Fab-8 sequence (**B**) bicoid sequence (**C**) CG31472 sequence (**D**) Fab-6 sequence (**E**) control site to compare general peaks of the insulator binding proteins (mb, mega base).
(TIF)

Figure S8 **Western blot after knockdown of CTCF plus CP190 and of ISWI demonstrates depletion of these factors.** S2 cells were transfected with dsRNA corresponding to dCTCF and CP190 (dsCTCF/CP190), ISWI (dsISWI) or firefly luciferase (dsLuci) as control. Cell extracts of two independent experiments were analyzed by Western blot with antibodies directed against dCTCF, CP190, ISWI or tubulin as loading control.
(TIF)

Figure S9 **Depletion of CTCF/CP190 and ISWI interferes with nucleosome depletion at CP190 positive DNase I hypersensitive sites.** Cumulative representation of changes in H3- binding and MNase-protection as detected by H3 ChIP-seq and MNase-seq after depletion of CTCF/CP190 (green; DKD) or ISWI (orange). Data is shown as coverage for specific knock-down normalized to luciferase control knock-down (luci) after log2-transformation. Average effects are shown across DNase I hypersensitive sites (DHSs; mapped by (Arnold et al. 2013)) positive for CP190 binding (colored) or control DHSs devoid of significant CP190 binding (grey).
(TIF)

Figure S10 **Depletion of CTCF/CP190 and ISWI interferes with nucleosome depletion as determined by MNase digestion or by H3 ChIP.** Representation of changes in H3-binding (top) and MNase-protection (bottom) after MNase treatment and H3 ChIP in S2 cells treated with dsRNA against dCTCF and CP190 (dsCTCF/CP190; green), ISWI (dsISWI; orange) or against luciferase as control (dsLuci; black). All sites with increased MNase-protection and H3 binding after CTCF/CP190 depletion (positive sites) show a similar MNase-protection and H3 increase upon ISWI depletion. Non-responding sites after CTCF/CP190 depletion do not respond to ISWI depletion. Error bars indicate the standard deviation error of the mean of two independent experiments (p-values: *≤0.05, **≤0.01, ***≤0.001).
(TIF)

Figure S11 **Depletion of CTCF/CP190 and ISWI interferes with nucleosome depletion at CP190 binding site clusters 2 to 5 marked by NURF an DREAM binding.** Cumulative representation of changes in histone H3-binding after depletion of CTCF/CP190 (A: green/DKD) or ISWI (B: orange). Data was analyzed separately for CP190 binding sites clusters 1–6 identified in Fig. 3 and is shown as coverage of specific knock-down normalized to luciferase control knock-down (luci after log2-transformation). Average binding across control sites shifted +25 kb is shown in grey.
(TIF)

Table S1 **Z-score list of all dsRNA sequences with a z- score ≥2.** NURF- and dREAM components are highlighted in pink and blue.
(XLSX)

Table S2 **Sequences, localisation, size and other characteristics of tested sites in the reporter assays (top) and ChIP assays (bottom).** bp, base pairs; chr, chromosome; fwd., forward; re, restriction enzyme; rev., reverse; TES, transcriptional end site; TSS, transcriptional start site.
(XLSX)

Text S1 **Supplementary information on methods and references.**
(DOCX)

## References

[1] Gaszner M, Felsenfeld G (2006) Insulators: exploiting transcriptional and epigenetic mechanisms. Nature reviews Genetics 7: 703–713. 10.1038/nrg1925 16909129

[2] Herold M, Bartkuhn M, Renkawitz R (2012) CTCF: insights into insulator function during development. Development 139: 1045–1057. 22354838 10.1242/dev.065268

[3] Yang J, Corces VG (2012) Insulators, long-range interactions, and genome function. Current opinion in genetics & development 22: 86–92. 22265227 10.1016/j.gde.2011.12.007 PMC3337973

[4] Gaszner M, Vazquez J, Schedl P (1999) The Zw5 protein, a component of the scs chromatin domain boundary, is able to block enhancer-promoter interaction. Genes Dev 13: 2098–2107. 10465787 10.1101/gad.13.16.2098 PMC316952

[5] Roy S, Tan YY, Hart CM (2006) A genetic screen supports a broad role for the Drosophila insulator proteins BEAF-32A and BEAF-32B in maintaining patterns of gene expression. Mol Genet Genomics 277: 273–286. 17143631 10.1007/s00438-006-0187-8

[6] Zhao K, Hart CM, Laemmli UK (1995) Visualization of chromosomal domains with boundary element-associated factor BEAF-32. Cell 81: 879–889. 7781065 10.1016/0092-8674(95)90008-x

[7] Ohtsuki S, Levine M (1998) GAGA mediates the enhancer blocking activity of the eve promoter in the Drosophila embryo. Genes Dev 12: 3325–3330. 9808619 10.1101/gad.12.21.3325 PMC317233

[8] Parkhurst SM, Harrison DA, Remington MP, Spana C, Kelley RL, et al (1988) The Drosophila su(Hw) gene, which controls the phenotypic effect of the gypsy transposable element, encodes a putative DNA-binding protein. Genes Dev 2: 1205–1215. 2462523 10.1101/gad.2.10.1205

[9] Negre N, Brown CD, Shah PK, Kheradpour P, Morrison CA, et al (2010) A comprehensive map of insulator elements for the Drosophila genome. PloS Genet 6: e1000814. 20084099 10.1371/journal.pgen.1000814 PMC2797089

[10] Schwartz YB, Linder-Basso D, Kharchenko PV, Tolstorukov MY, Kim M, et al (2012) Nature and function of insulator protein binding sites in the Drosophila genome. Genome Research 22: 2188–2198. 22767387 10.1101/gr.138156.112 PMC3483548

[11] Van Bortle K, Ramos E, Takenaka N, Yang J, Wahi JE, et al (2012) Drosophila CTCF tandemly aligns with other insulator proteins at the borders of H3K27me3 domains. Genome Research 22: 2176–2187. 22722341 10.1101/gr.136788.111 PMC3483547

[12] Bartkuhn M, Straub T, Herold M, Herrmann M, Rathke C, et al (2009) Active promoters and insulators are marked by the centrosomal protein 190. Embo J 28: 877–888. 19229299 10.1038/emboj.2009.34 PMC2670862

[13] Ahanger SH, Shouche YS, Mishra RK (2013) Functional sub-division of the Drosophila genome via chromatin looping: The emerging importance of CP190. Nucleus 4: 115–122. 23333867 10.4161/nucl.23389 PMC3621743

[14] Li HB, Ohno K, Gui H, Pirrotta V (2013) Insulators target active genes to transcription factories and polycomb-repressed genes to polycomb bodies. PLoS genetics 9: e1003436. 23637616 10.1371/journal.pgen.1003436 PMC3630138

[15] Barges S, Mihaly J, Galloni M, Hagstrom K, Muller M, et al (2000) The Fab-8 boundary defines the distal limit of the bithorax complex iab-7 domain and insulates iab-7 from initiation elements and a PRE in the adjacent iab-8 domain. Development 127: 779–790. 10648236 10.1242/dev.127.4.779

[16] Lewis EB (1978) A gene complex controlling segmentation in Drosophila. Nature 276: 565–570. 103000 10.1038/276565a0

[17] Holohan EE, Kwong C, Adryan B, Bartkuhn M, Herold M, et al (2007) CTCF Genomic Binding Sites in Drosophila and the Organisation of the Bithorax Complex. PloS Genet 3: e112. 17616980 10.1371/journal.pgen.0030112 PMC1904468

[18] Mohan M, Bartkuhn M, Herold M, Philippen A, Heinl N, et al (2007) The Drosophila insulator proteins CTCF and CP190 link enhancer blocking to body patterning. Embo J 26: 4203–4214. 17805343 10.1038/sj.emboj.7601851 PMC2230845

[19] Gerasimova TI, Lei EP, Bushey AM, Corces VG (2007) Coordinated control of dCTCF and gypsy chromatin insulators in Drosophila. Mol Cell 28: 761–772. 18082602 10.1016/j.molcel.2007.09.024 PMC2579779

[20] Ciavatta D, Rogers S, Magnuson T (2007) Drosophila CTCF is required for Fab-8 enhancer blocking activity in S2 cells. Journal of Molecular Biology 373: 233–239. 17825318 10.1016/j.jmb.2007.07.065 PMC2694738

[21] Tsukiyama T, Daniel C, Tamkun J, Wu C (1995) ISWI, a member of the SWI2/SNF2 ATPase family, encodes the 140 kDa subunit of the nucleosome remodeling factor. Cell 83: 1021–1026. 8521502 10.1016/0092-8674(95)90217-1

[22] Clapier CR, Cairns BR (2009) The biology of chromatin remodeling complexes. Annual review of biochemistry 78: 273–304. 10.1146/annurev.biochem.77.062706.153223 19355820

[23] Korenjak M, Taylor-Harding B, Binné UK, Satterlee JS, Stevaux O, et al (2004) Native E2F/RBF complexes contain Myb-interacting proteins and repress transcription of developmentally controlled E2F target genes. Cell 119: 181–193. 15479636 10.1016/j.cell.2004.09.034

[24] Lewis PW, Beall EL, Fleischer TC, Georlette D, Link AJ, et al (2004) Identification of a Drosophila Myb-E2F2/RBF transcriptional repressor complex. Genes & Development 18: 2929–2940. 15545624 10.1101/gad.1255204 PMC534653

[25] van den Heuvel S, Dyson NJ (2008) Conserved functions of the pRB and E2F families. Nat Rev Mol Cell Biol 9: 713–724. 18719710 10.1038/nrm2469

[26] Kugler SJ, Nagel AC (2010) A novel Pzg-NURF complex regulates Notch target gene activity. Molecular biology of the cell 21: 3443–3448. 20685964 10.1091/mbc.E10-03-0212 PMC2947479

[27] Hochheimer A, Zhou S, Zheng S, Holmes MC, Tjian R (2002) TRF2 associates with DREF and directs promoter-selective gene expression in Drosophila. Nature 420: 439–445. 12459787 10.1038/nature01167

[28] Gurudatta BV, Yang J, Van Bortle K, Donlin-Asp PG, Corces VG (2013) Dynamic changes in the genomic localization of DNA replication-related element binding factor during the cell cycle. Cell cycle 12. 10.4161/cc.24742 PMC3680540 23624840

[29] Beall EL, Manak JR, Zhou S, Bell M, Lipsick JS, et al (2002) Role for a Drosophila Myb-containing protein complex in site-specific DNA replication. Nature 420: 833–837. 12490953 10.1038/nature01228

[30] Martinez-Balbas MA, Tsukiyama T, Gdula D, Wu C (1998) Drosophila NURF-55, a WD repeat protein involved in histone metabolism. Proceedings of the National Academy of Sciences of the United States of America 95: 132–137. 9419341 10.1073/pnas.95.1.132 PMC18150

[31] Feller C, Prestel M, Hartmann H, Straub T, Soding J, et al (2012) The MOF-containing NSL complex associates globally with housekeeping genes, but activates only a defined subset. Nucleic Acids Research 40: 1509–1522. 22039099 10.1093/nar/gkr869 PMC3287193

[32] Roy S, Ernst J, Kharchenko PV, Kheradpour P, Negre N, et al (2010) Identification of functional elements and regulatory circuits by Drosophila modENCODE. Science 330: 1787–1797. 21177974 10.1126/science.1198374 PMC3192495

[33] Georlette D, Ahn S, MacAlpine DM, Cheung E, Lewis PW, et al (2007) Genomic profiling and expression studies reveal both positive and negative activities for the Drosophila Myb MuvB/dREAM complex in proliferating cells. Genes & Development 21: 2880–2896. 17978103 10.1101/gad.1600107 PMC2049191

[34] Kyrchanova O, Ivlieva T, Toshchakov S, Parshikov A, Maksimenko O, et al (2011) Selective interactions of boundaries with upstream region of Abd-B promoter in Drosophila bithorax complex and role of dCTCF in this process. Nucleic Acids Res 39: 3042–3052. 21149269 10.1093/nar/gkq1248 PMC3082887

[35] Smith ST, Wickramasinghe P, Olson A, Loukinov D, Lin L, et al (2009) Genome wide ChIP-chip analyses reveal important roles for CTCF in Drosophila genome organization. Dev Biol 328: 518–528. 19210964 10.1016/j.ydbio.2008.12.039 PMC6620017

[36] Perez-Lluch S, Cuartero S, Azorin F, Espinas ML (2008) Characterization of new regulatory elements within the Drosophila bithorax complex. Nucleic Acids Res 36: 6926–6933. 18978017 10.1093/nar/gkn818 PMC2588531

[37] Arnold CD, Gerlach D, Stelzer C, Boryn LM, Rath M, et al (2013) Genome-wide quantitative enhancer activity maps identified by STARR-seq. Science 339: 1074–1077. 23328393 10.1126/science.1232542

[38] Wen H, Andrejka L, Ashton J, Karess R, Lipsick JS (2008) Epigenetic regulation of gene expression by Drosophila Myb and E2F2-RBF via the Myb-MuvB/dREAM complex. Genes & Development 22: 601–614. 18316477 10.1101/gad.1626308 PMC2259030

[39] Lee H, Ohno K, Voskoboynik Y, Ragusano L, Martinez A, et al (2010) Drosophila RB proteins repress differentiation-specific genes via two different mechanisms. Molecular and Cellular Biology 30: 2563–2577. 20176807 10.1128/MCB.01075-09 PMC2863701

[40] Lee H, Ragusano L, Martinez A, Gill J, Dimova DK (2012) A dual Role for the dREAM/MMB complex in the regulation of differentiation-specific E2F/RB target genes. Molecular and Cellular Biology. 10.1128/MCB.06314-11 PMC3372228 22451490

[41] Macaluso M, Montanari M, Giordano A (2006) Rb family proteins as modulators of gene expression and new aspects regarding the interaction with chromatin remodeling enzymes. Oncogene 25: 5263–5267. 16936746 10.1038/sj.onc.1209680

[42] Frolov MV, Dyson NJ (2004) Molecular mechanisms of E2F-dependent activation and pRB-mediated repression. Journal of cell science 117: 2173–2181. 15126619 10.1242/jcs.01227

[43] Sim CK, Perry S, Tharadra SK, Lipsick JS, Ray A (2012) Epigenetic regulation of olfactory receptor gene expression by the Myb-MuvB/dREAM complex. Genes & Development 26: 2483–2498. 23105004 10.1101/gad.201665.112 PMC3505819

[44] Li M, Belozerov VE, Cai HN (2010) Modulation of chromatin boundary activities by nucleosome-remodeling activities in Drosophila melanogaster. Molecular and Cellular Biology 30: 1067–1076. 19995906 10.1128/MCB.00183-09 PMC2815568

[45] Wendt KS, Yoshida K, Itoh T, Bando M, Koch B, et al (2008) Cohesin mediates transcriptional insulation by CCCTC-binding factor. Nature 451: 796–801. 18235444 10.1038/nature06634

[46] Holwerda SJ, de Laat W (2013) CTCF: the protein, the binding partners, the binding sites and their chromatin loops. Philosophical transactions of the Royal Society of London Series B, Biological sciences 368: 20120369. 23650640 10.1098/rstb.2012.0369 PMC3682731

[47] DeMare LE, Leng J, Cotney J, Reilly SK, Yin J, et al (2013) The genomic landscape of cohesin-associated chromatin interactions. Genome Research 23: 1224–1234. 23704192 10.1101/gr.156570.113 PMC3730097

[48] Ong CT, Van Bortle K, Ramos E, Corces VG (2013) Poly(ADP-ribosyl)ation Regulates Insulator Function and Intrachromosomal Interactions in Drosophila. Cell 155: 148–159. 24055367 10.1016/j.cell.2013.08.052 PMC3816015

[49] Phillips-Cremins JE, Sauria ME, Sanyal A, Gerasimova TI, Lajoie BR, et al (2013) Architectural protein subclasses shape 3D organization of genomes during lineage commitment. Cell 153: 1281–1295. 23706625 10.1016/j.cell.2013.04.053 PMC3712340

[50] Raab JR, Kamakaka RT (2010) Insulators and promoters: closer than we think. Nat Rev Genet 11: 439–446. 20442713 10.1038/nrg2765 PMC3477808

[51] Bai L, Morozov AV (2010) Gene regulation by nucleosome positioning. Trends in genetics: TIG 26: 476–483. 20832136 10.1016/j.tig.2010.08.003

[52] Schoenfelder S, Clay I, Fraser P (2010) The transcriptional interactome: gene expression in 3D. Current opinion in genetics & development 20: 127–133. 20211559 10.1016/j.gde.2010.02.002

[53] Yusufzai TM, Tagami H, Nakatani Y, Felsenfeld G (2004) CTCF tethers an insulator to subnuclear sites, suggesting shared insulator mechanisms across species. Mol Cell 13: 291–298. 14759373 10.1016/s1097-2765(04)00029-2

[54] Frasch M, Glover DM, Saumweber H (1986) Nuclear antigens follow different pathways into daughter nuclei during mitosis in early Drosophila embryos. J Cell Sci 82: 155–172. 3098744 10.1242/jcs.82.1.155

[55] Moshkin YM, Chalkley GE, Kan TW, Reddy BA, Ozgur Z, et al (2012) Remodelers organize cellular chromatin by counteracting intrinsic histone-DNA sequence preferences in a class-specific manner. Mol Cell Biol 32: 675–688. 22124157 10.1128/MCB.06365-11 PMC3266603

[56] Gan M, Moebus S, Eggert H, Saumweber H (2011) The Chriz-Z4 complex recruits JIL-1 to polytene chromosomes, a requirement for interband-specific phosphorylation of H3S10. J Biosci 36: 425–438. 21799255 10.1007/s12038-011-9089-y

[57] Tyler JK, Bulger M, Kamakaka RT, Kobayashi R, Kadonaga JT (1996) The p55 subunit of Drosophila chromatin assembly factor 1 is homologous to a histone deacetylase-associated protein. Mol Cell Biol 16: 6149–6159. 8887645 10.1128/mcb.16.11.6149 PMC231618

[58] Carmona-Saez P, Chagoyen M, Tirado F, Carazo JM, Pascual-Montano A (2007) GENECODIS: a web-based tool for finding significant concurrent annotations in gene lists. Genome Biol 8: R3. 17204154 10.1186/gb-2007-8-1-r3 PMC1839127

[59] Nogales-Cadenas R, Carmona-Saez P, Vazquez M, Vicente C, Yang X, et al (2009) GeneCodis: interpreting gene lists through enrichment analysis and integration of diverse biological information. Nucleic Acids Res 37: W317–322. 19465387 10.1093/nar/gkp416 PMC2703901

[60] Tabas-Madrid D, Nogales-Cadenas R, Pascual-Montano A (2012) GeneCodis3: a non-redundant and modular enrichment analysis tool for functional genomics. Nucleic Acids Res 40: W478–483. 22573175 10.1093/nar/gks402 PMC3394297

