# Supplementary figures and images for: A Functional Insulator Screen Identifies NURF and dREAM Components to Be Required for Enhancer-Blocking
Source: PLoS One. 2014 Sep 23;9(9):e107765. doi: 10.1371/journal.pone.0107765 (PMC4172637; doi:10.1371/journal.pone.0107765)

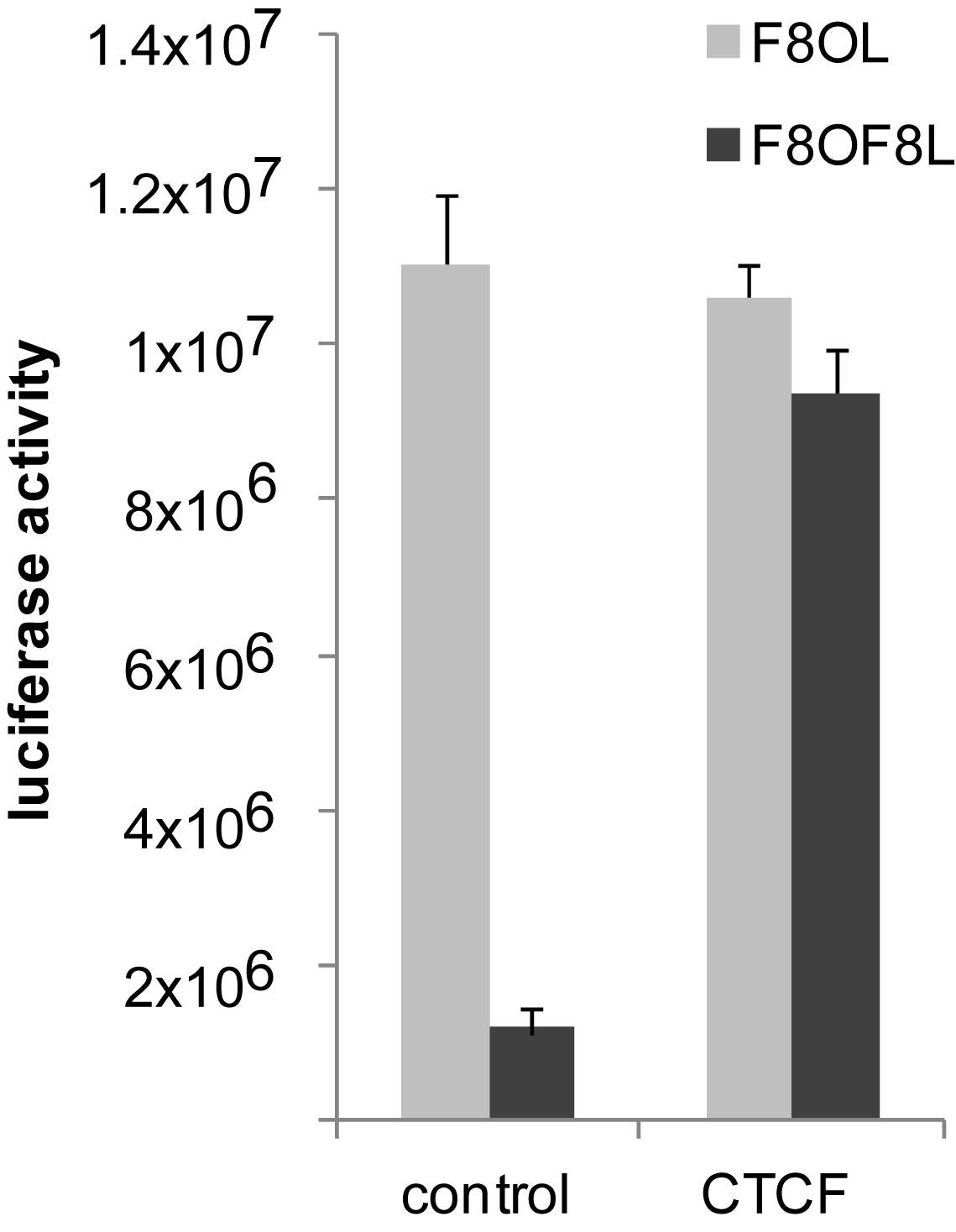

Supplement: Figure S1 — F8OL and F8OF8L clone pools show the expected CTCF dependent enhancer blocking. S2 cell clone pools with the integrated F8OF8L insulator reporter or the control F8OL reporter construct were incubated with dsRNA against GFP (control) or against CTCF (CTCF). Reporter gene activity is expressed as relative light units. Error bars indicate the standard deviation of three individual replicates. (TIF) [file pone.0107765.s001.tif]

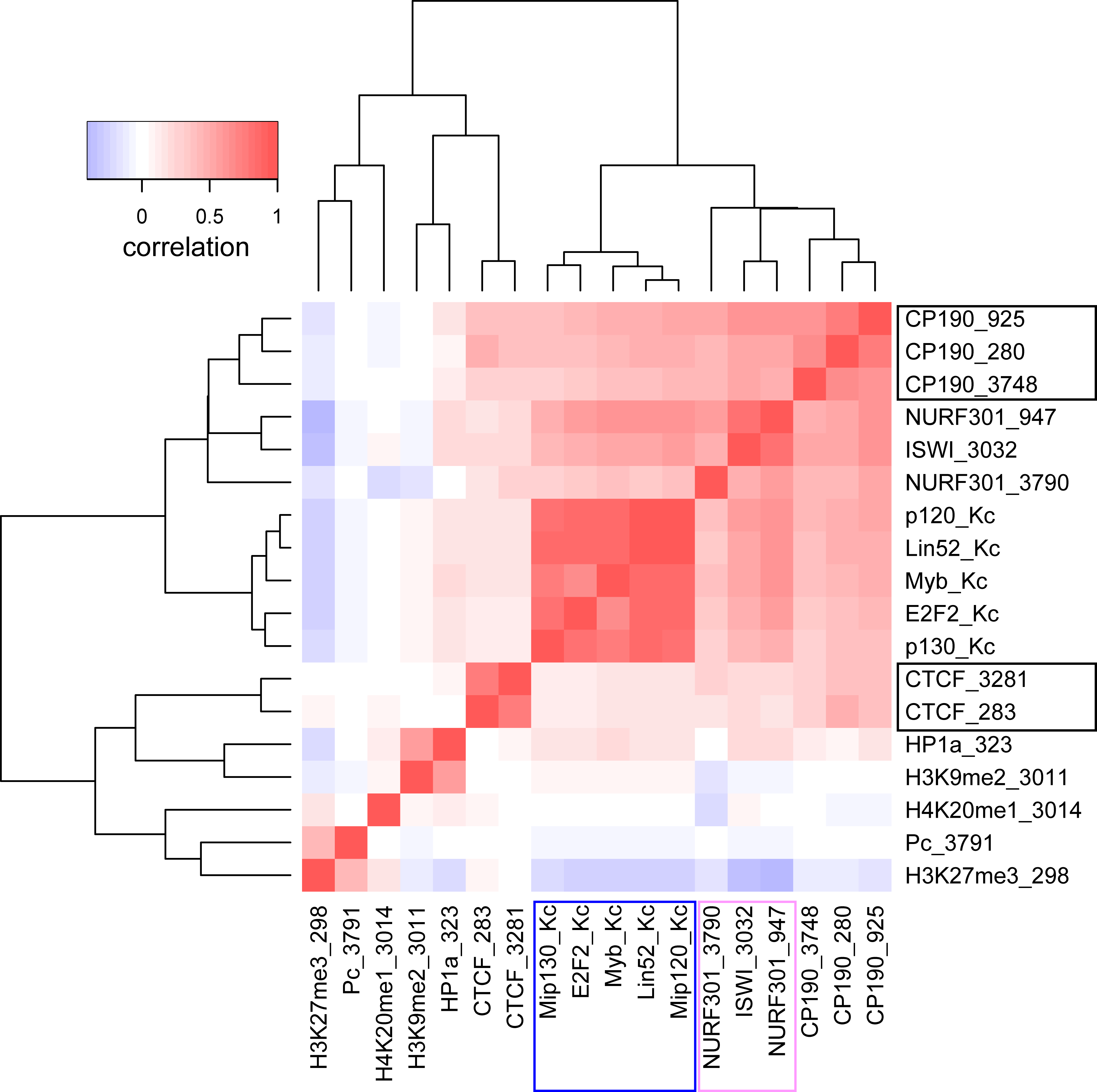

Supplement: Figure S2 — CP190 binding profiles are very similar to DREAM and ISWI/Nurf301 profiles. Publicly available ChIP-chip data for DREAM components (Mip130, Mip120, E2F2, Myb, Lin-52) (Georlette et al. 2007) as well as CP190, CTCF, ISWI, Nurf301 and several other profiles (ModEncode) serving as controls were binned into 100 bp bins by calculating the average enrichment of ChIP over input within each bin. Pair wise correlation coefficients were calculated. Hierarchical clustering of coefficients is shown as color coded heat map. The numbers behind ModEncode derived profile names refer to ModEncode IDs. (TIF) [file pone.0107765.s002.tif]

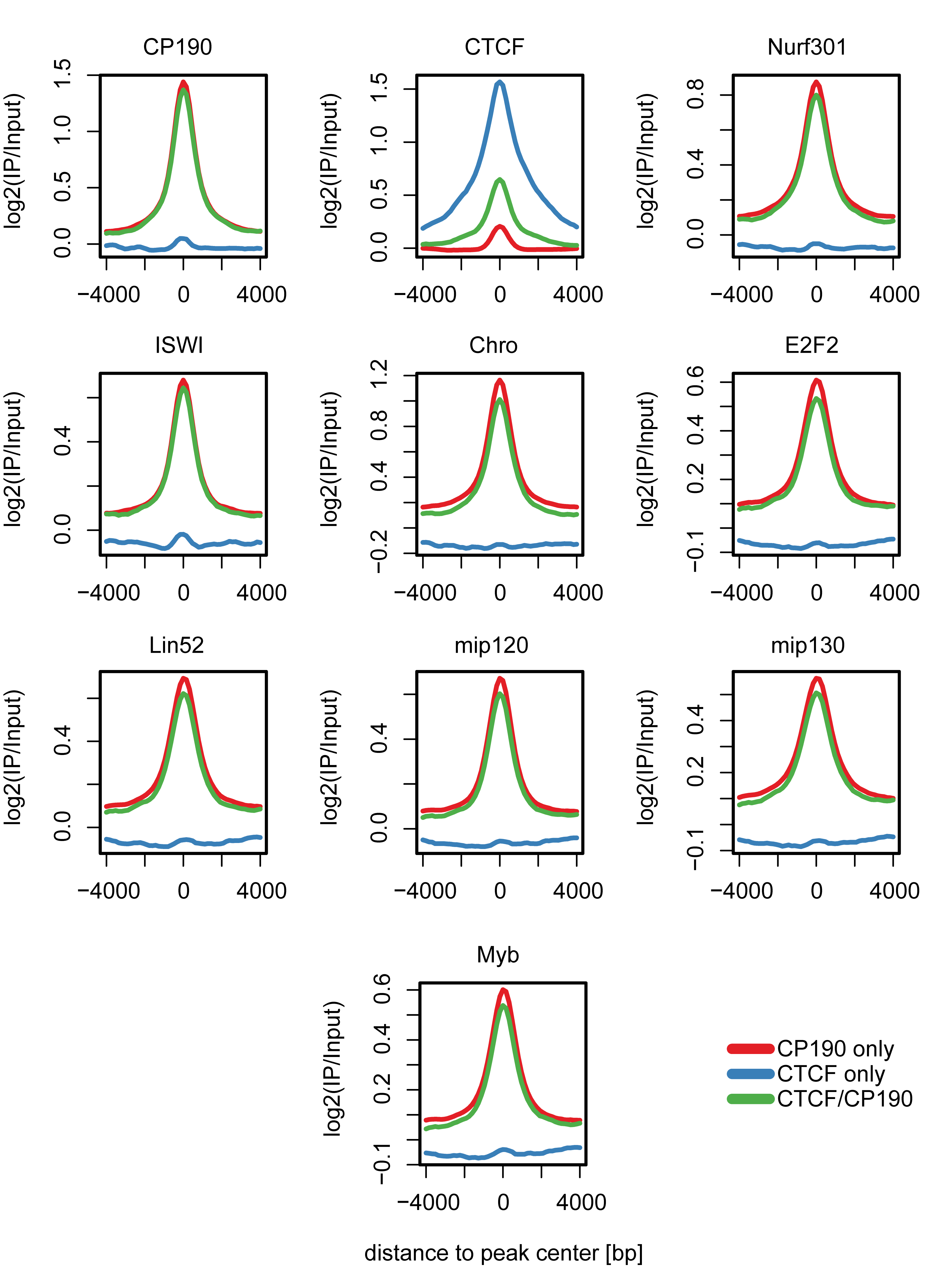

Supplement: Figure S3 — DREAM and NURF associate with CP190 and CTCF/CP190 but not with stand-alone CTCF binding sites. Cumulative binding profiles for indicated factors across the 3 classes of CTCF/CP190 binding sites (CTCF only, CP190 only and common CTCF/CP190). Stand-alone CP190 as well as common CTCF/CP190 sites are bound by DREAM and NURF components to a similar extent whereas stand-alone CTCF sites are devoid of both complexes. All binding data are from ModENCODE. (TIF) [file pone.0107765.s003.tif]

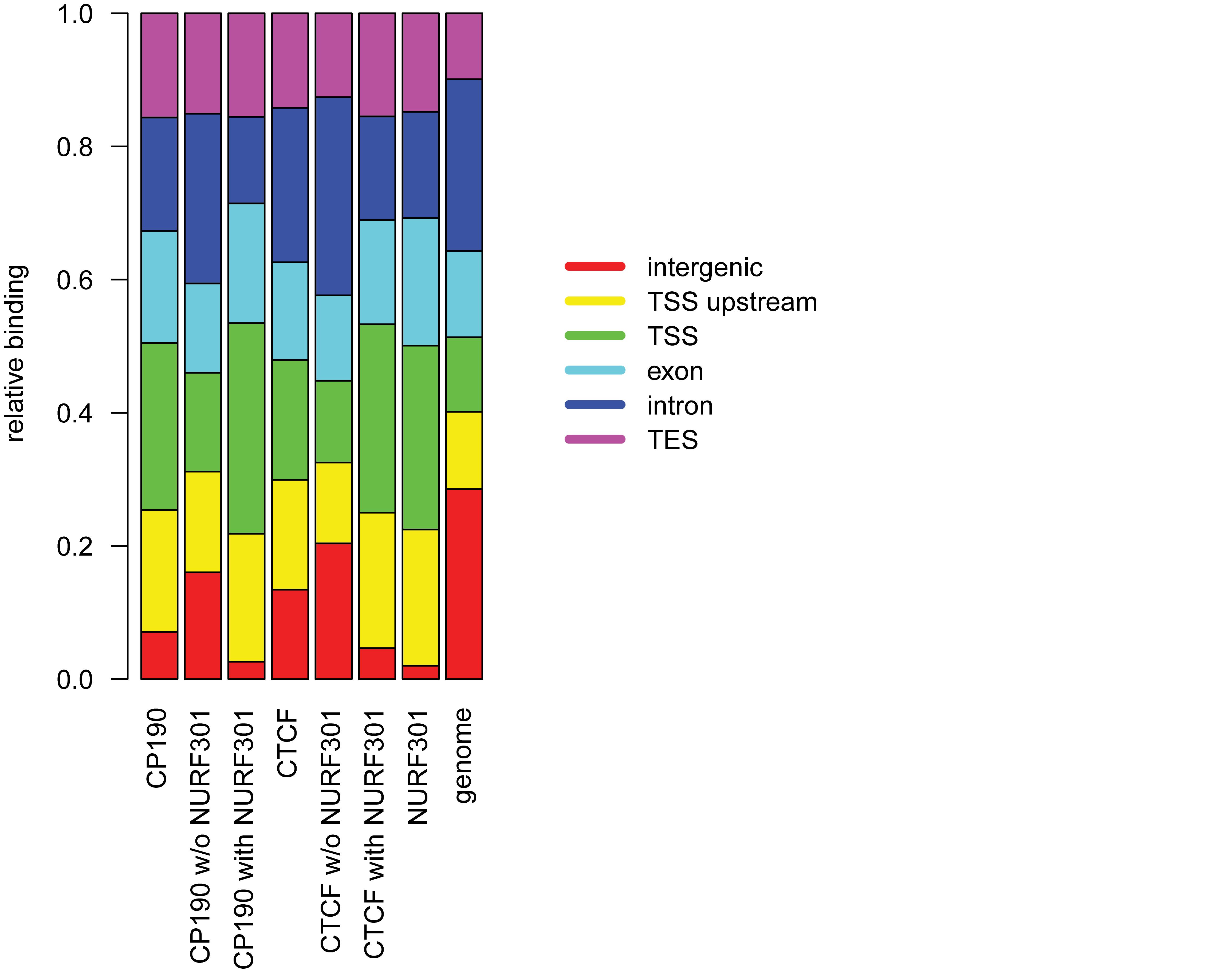

Supplement: Figure S4 — CTCF as well as CP190 sites bound simultaneously by NURF are enriched for promotor associated annotations. Distribution of genomic elements (red for intergenic, yellow for transcriptional start site (TSS) upstream region (−1 kb to −10 kb upstream of TSS), green for TSS (+/−1 kb around TSS), light blue for exon and dark blue for intron and purple for transcriptional end sites (TES)) across CTCF and CP190 binding sites with respect to overlap with NURF301 binding (data from ModENCODE). Enrichment for TSS-associated binding of CTCF and CP190 is associated with simultaneous NURF301 binding. (TIF) [file pone.0107765.s004.tif]

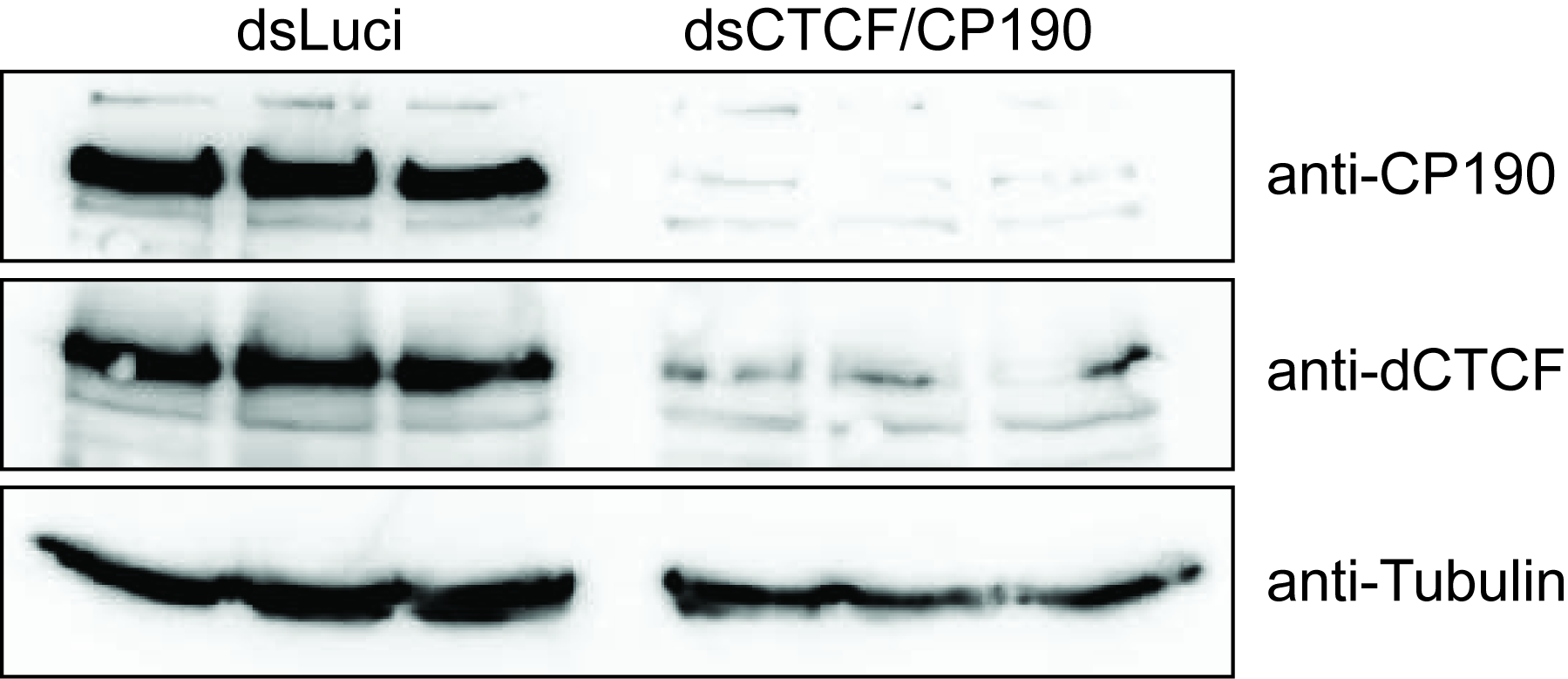

Supplement: Figure S5 — Western blot after knockdown of CTCF and CP190 demonstrates CP190 and CTCF depletion. S2 cells were transfected with dsRNA corresponding to dCTCF and CP190 (dsCTCF/CP190) or firefly luciferase (dsLuci) as control. Cell extracts of three independent experiments were analyzed by Western blot with antibodies directed against dCTCF, CP190 or tubulin as loading control. (TIF) [file pone.0107765.s005.tif]

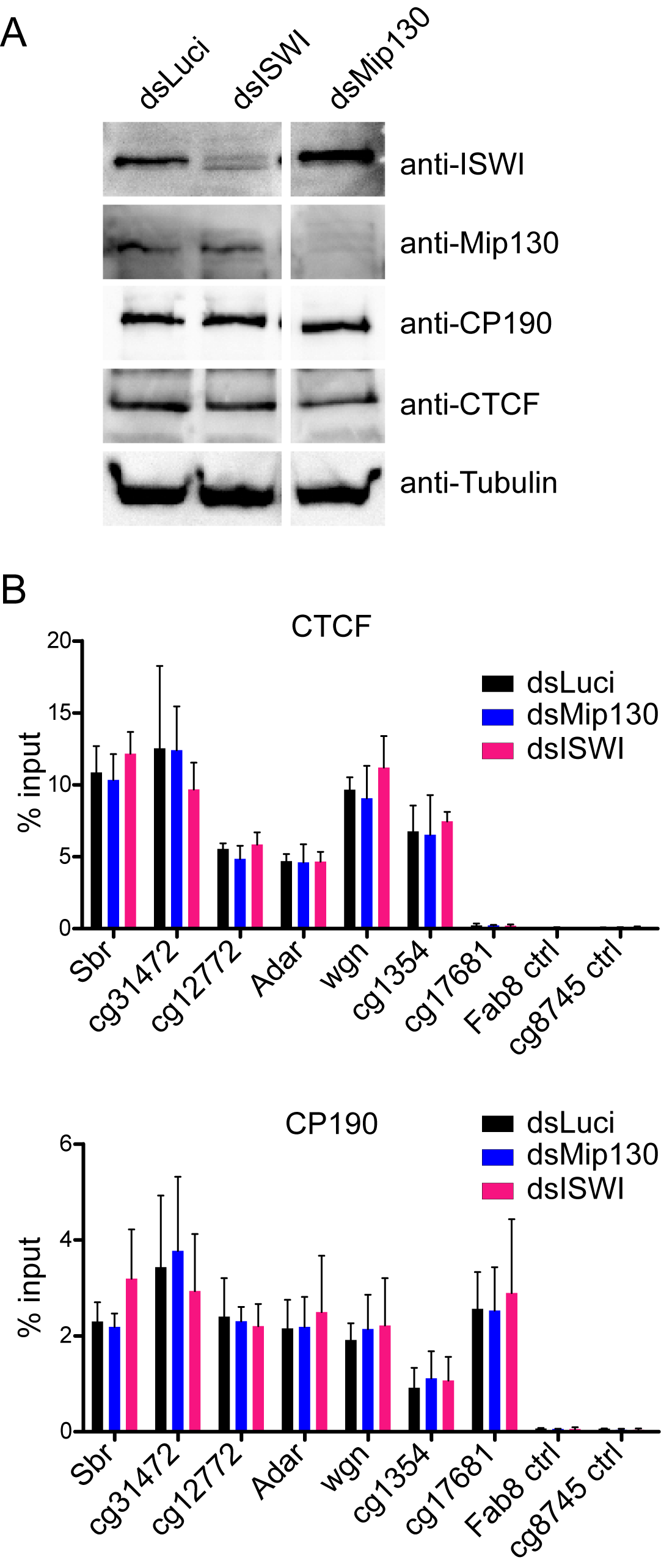

Supplement: Figure S6 — Depletion of ISWI or MIP130 does not affect CTCF or CP190 binding. (A) Western blot after knockdown of ISWI (dsISWI; NURF complex) and Mip130 (dsMip130; dREAM complex) demonstrates ISWI and Mip130 depletion, but no influence on CTCF/CP190 protein level. knockdown control, dsLuci; protein loading control, Tubulin. (B) ChIP in S2 cells treated with dsRNA against ISWI (dsISWI) and Mip130 (dsMip130) or against luciferase as control (dsLuci). Antibodies were used specific for dCTCF (top) and CP190 (bottom). The genomic regions tested are strong binding sites for dCTCF and CP190: Sbr, cg31472, Adar, cg12772, wgn, CG1354; a weak binding site for dCTCF: cg17681 and two negative control sites: Fab-8 ctrl and cg8745 ctrl. Values are expressed as % input. Error bars indicate the standard deviation of three independent experiments. (TIF) [file pone.0107765.s006.tif]

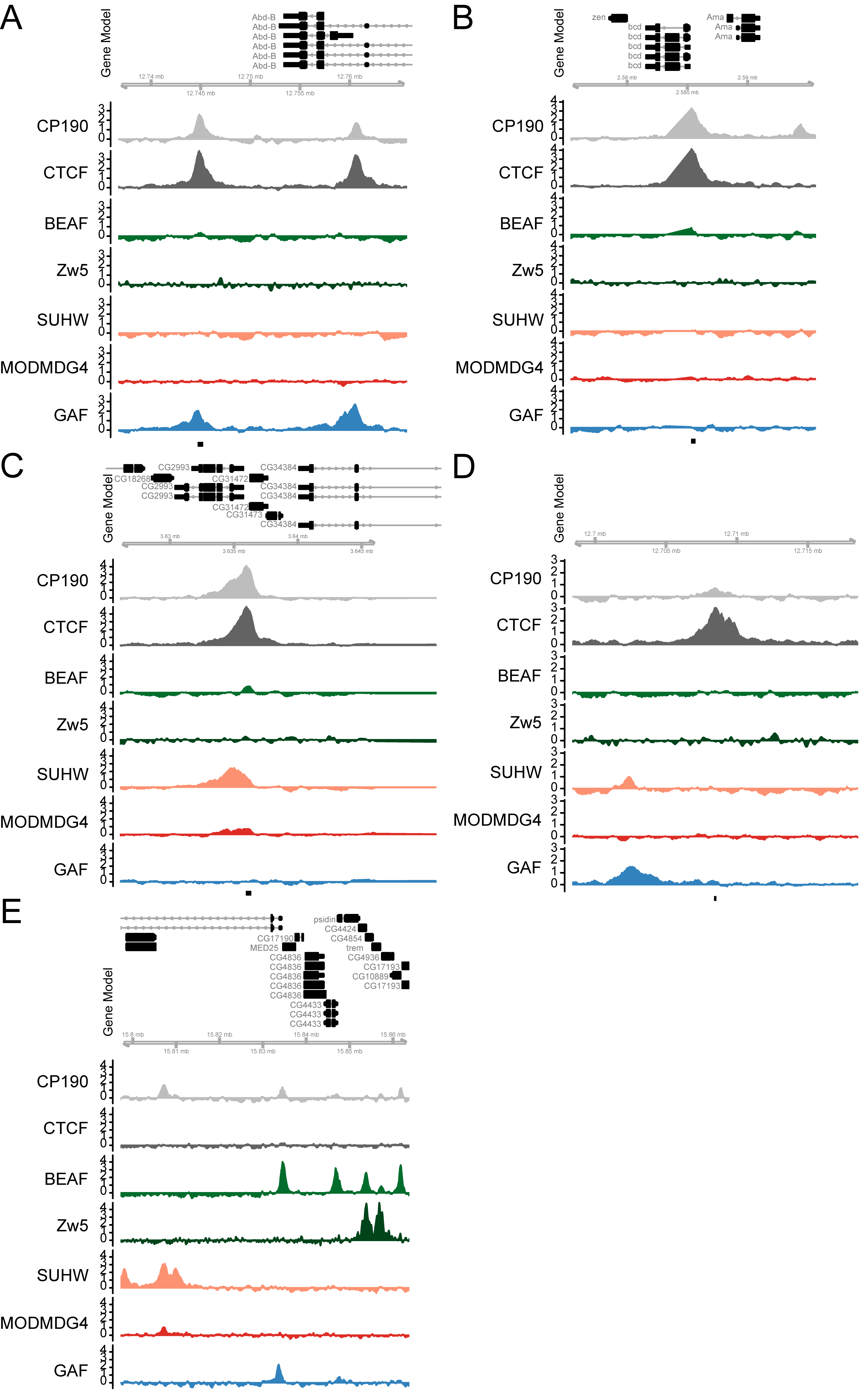

Supplement: Figure S7 — Genome browser view of insulators Fab-8, bcd, CG31472 and Fab-6. Publicly available ChIP-chip data for CP190, CTCF and other insulator binding proteins (BEAF, Zw5, Su(Hw), Modmdg4 and GAF) (ModEncode) show the binding profiles at the tested insulator elements (bottom black box in each case). Known transcripts are shown at the top in each case. (A) Fab-8 sequence (B) bicoid sequence (C) CG31472 sequence (D) Fab-6 sequence (E) control site to compare general peaks of the insulator binding proteins (mb, mega base). (TIF) [file pone.0107765.s007.tif]

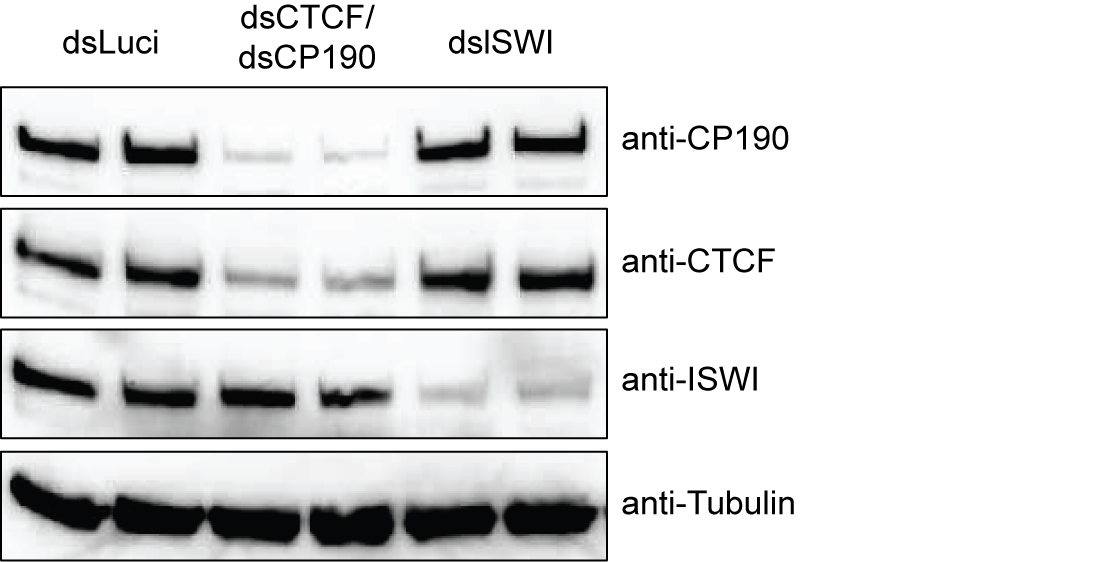

Supplement: Figure S8 — Western blot after knockdown of CTCF plus CP190 and of ISWI demonstrates depletion of these factors. S2 cells were transfected with dsRNA corresponding to dCTCF and CP190 (dsCTCF/CP190), ISWI (dsISWI) or firefly luciferase (dsLuci) as control. Cell extracts of two independent experiments were analyzed by Western blot with antibodies directed against dCTCF, CP190, ISWI or tubulin as loading control. (TIF) [file pone.0107765.s008.tif]

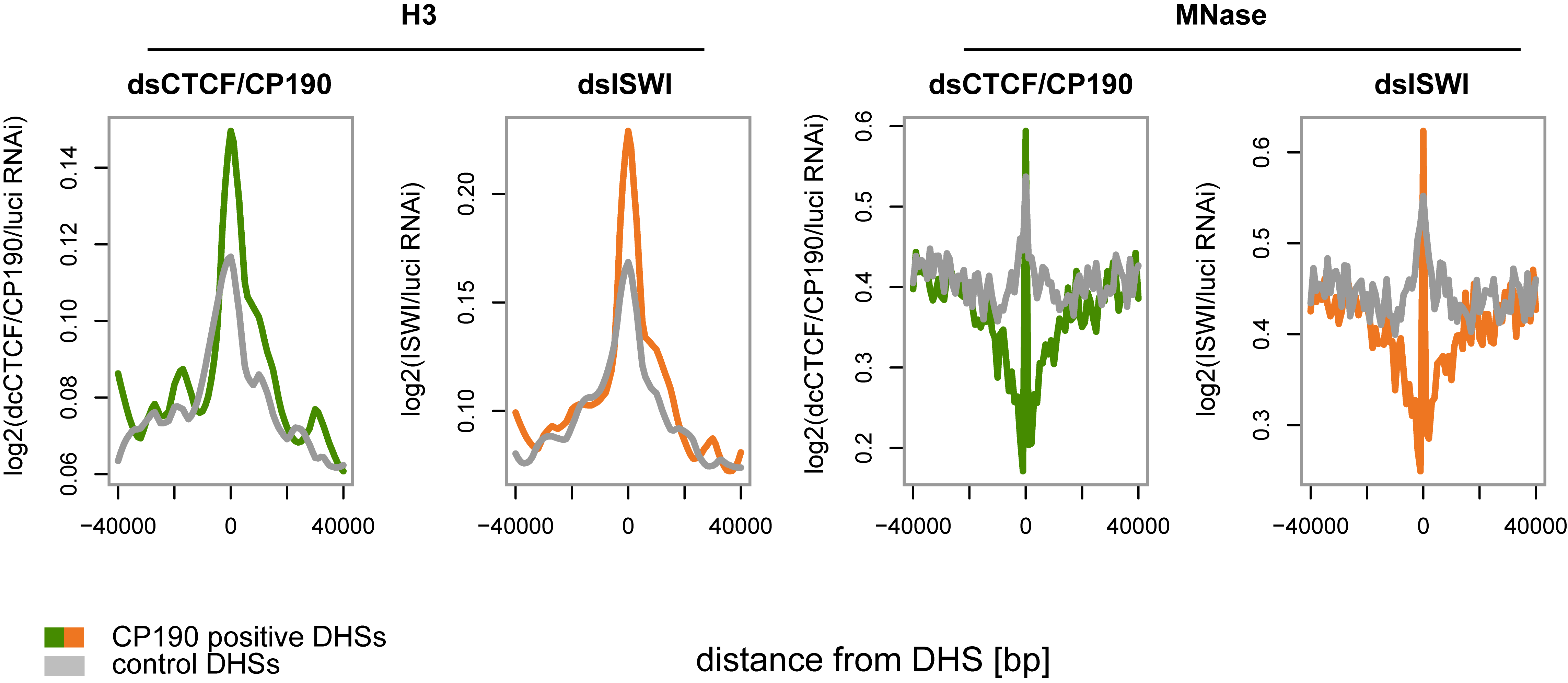

Supplement: Figure S9 — Depletion of CTCF/CP190 and ISWI interferes with nucleosome depletion at CP190 positive DNase I hypersensitive sites. Cumulative representation of changes in H3- binding and MNase-protection as detected by H3 ChIP-seq and MNase-seq after depletion of CTCF/CP190 (green; DKD) or ISWI (orange). Data is shown as coverage for specific knock-down normalized to luciferase control knock-down (luci) after log2-transformation. Average effects are shown across DNase I hypersensitive sites (DHSs; mapped by (Arnold et al. 2013)) positive for CP190 binding (colored) or control DHSs devoid of significant CP190 binding (grey). (TIF) [file pone.0107765.s009.tif]

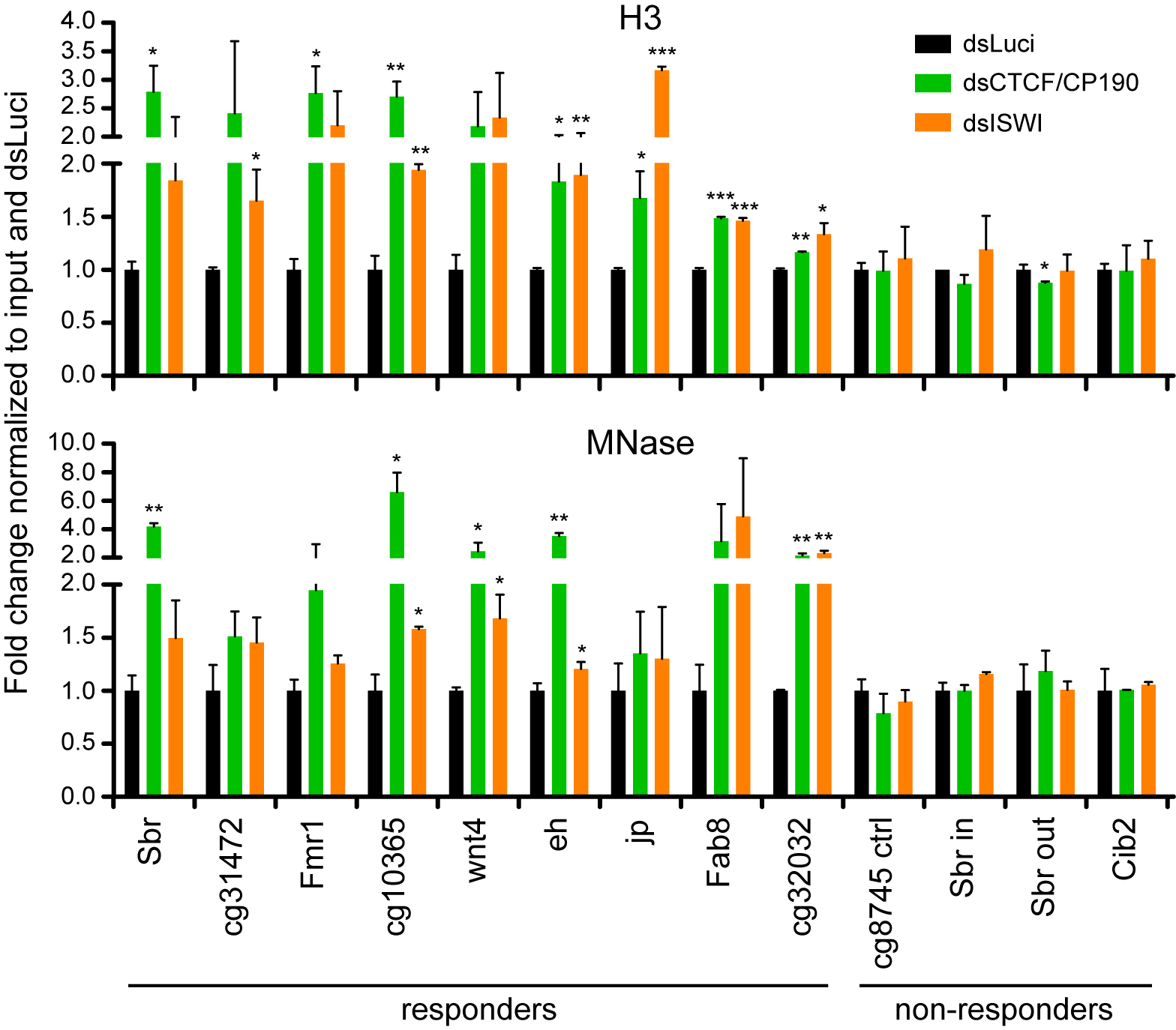

Supplement: Figure S10 — Depletion of CTCF/CP190 and ISWI interferes with nucleosome depletion as determined by MNase digestion or by H3 ChIP. Representation of changes in H3-binding (top) and MNase-protection (bottom) after MNase treatment and H3 ChIP in S2 cells treated with dsRNA against dCTCF and CP190 (dsCTCF/CP190; green), ISWI (dsISWI; orange) or against luciferase as control (dsLuci; black). All sites with increased MNase-protection and H3 binding after CTCF/CP190 depletion (positive sites) show a similar MNase-protection and H3 increase upon ISWI depletion. Non-responding sites after CTCF/CP190 depletion do not respond to ISWI depletion. Error bars indicate the standard deviation error of the mean of two independent experiments (p-values: *≤0.05, **≤0.01, ***≤0.001). (TIF) [file pone.0107765.s010.tif]

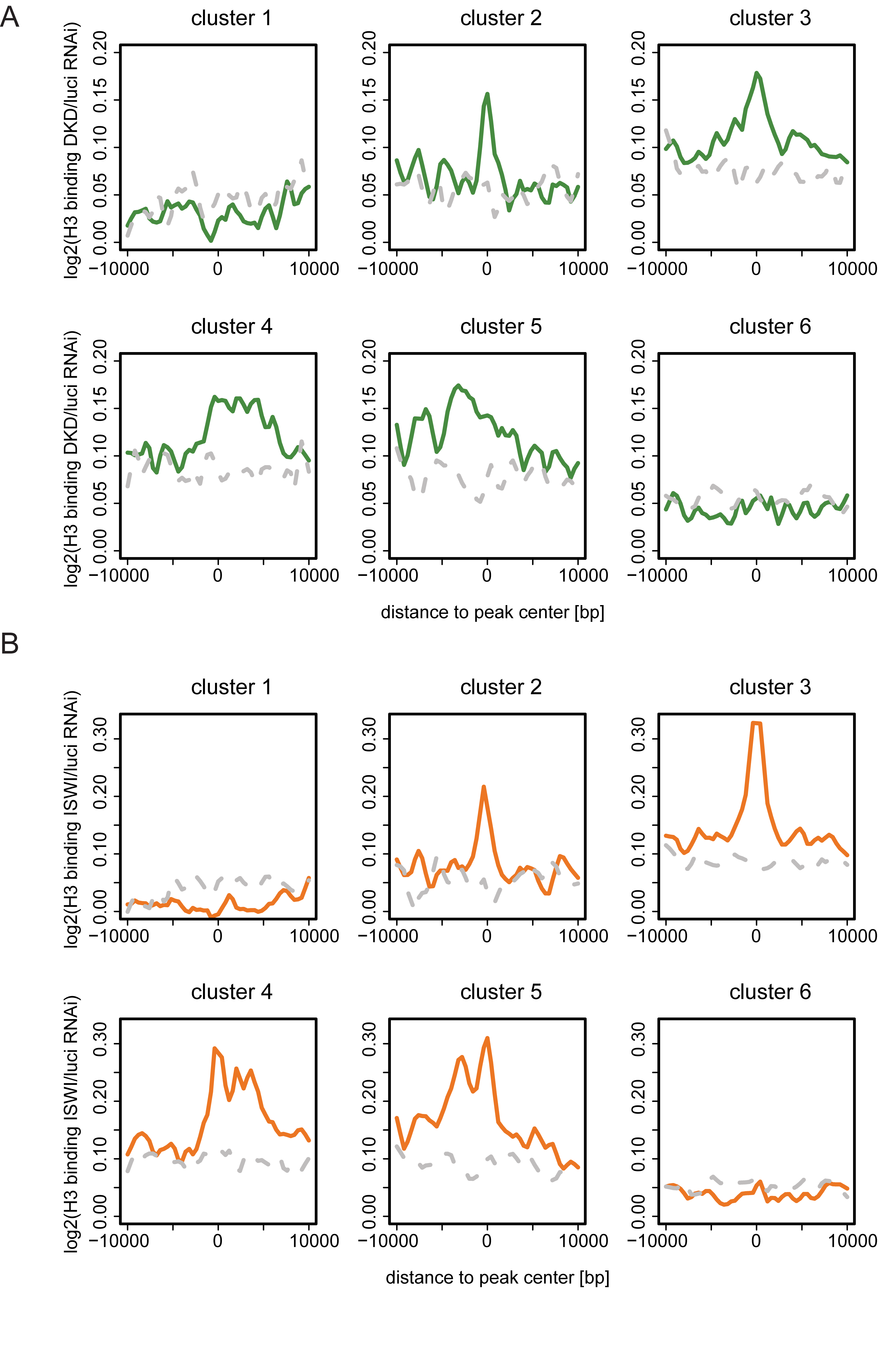

Supplement: Figure S11 — Depletion of CTCF/CP190 and ISWI interferes with nucleosome depletion at CP190 binding site clusters 2 to 5 marked by NURF an DREAM binding. Cumulative representation of changes in histone H3-binding after depletion of CTCF/CP190 (A: green/DKD) or ISWI (B: orange). Data was analyzed separately for CP190 binding sites clusters 1–6 identified in Fig. 3 and is shown as coverage of specific knock-down normalized to luciferase control knock-down (luci after log2-transformation). Average binding across control sites shifted +25 kb is shown in grey. (TIF) [file pone.0107765.s011.tif]
