# Supplementary material for: A Functional Insulator Screen Identifies NURF and dREAM Components to Be Required for Enhancer-Blocking
Source: PLoS One. 2014 Sep 23;9(9):e107765. doi: 10.1371/journal.pone.0107765 (PMC4172637; doi:10.1371/journal.pone.0107765)
Supplement: Text S1 — Supplementary information on methods and references. (DOCX) [file pone.0107765.s014.docx]

**File S1. Supplementary information on methods, primers and references**

**Methods**

*RNAi screening*

384-well plates with pre-aliquoted dsRNA (0.25µg/5 µl/well) (DRSC 2.0from the Drosophila RNAi Screening Center at Harvard Medical School (http://www.flyrnai.org/)) were thawed for a few minutes and spun at ~600g for 1 min. The S2F8OF8L clone pool was harvested from a confluent flask and collected in a 50-ml falcon by centrifugation at ~100g for 10 min twice, including a washing step. The cell pellet was resuspended at 1.5x10^6^ cells/ml in serum free Schneider’s medium (Invitrogen; supplemented with 1% penicillin/streptomycin and glutamine). The adhesive seal on the plates was removed and control dsRNA against CTCF was loaded into two defined empty wells (0.25µg/5µl/well), available at the same position of each plate. 10 µl of the cells were uniformly dispensed into all wells using a 96-well Manual Benchtop Pipettor (Liquidator™ 96 from Mettler Toledo). The plates were incubated and tilted twice for 45 min at 25°C. After incubation 30 µl of complete Schneider’s medium was added to each well. 10 plates were processed in one set of experiments. The plates were sealed and incubated at 25°Cfor 4 days. At day 4, 22μl of 3-fold concentrated lysis buffer was added with a multichannel pipette without removing the culture medium and plates were tilted at room temperature for 15 min. The luciferase activity was measured in a 384-well luminometer (Infinite® 200 PRO from Tecan) by injecting 22µl of luciferin into each well. The data was sent to the DRSC with the exact attribution of the content of each well to analyze the z-score of each factor.

*RNAi hit validation*

The experiments were done in 96- and 6-well plates, triplicates were carried out for controls and all RNAi treatments. Controls were either without dsRNA or GFP-specific dsRNA resulting in both cases in no effect on the screen cell pool S2F8OF8L or the control cell pool S2F8OL. Clone pools were harvested from the confluent flask and collected each in a 50-ml falcon by centrifugation at ~100 g for 10 min twice, including a washing step. 96-well-plates: The cell pellet was resuspended at 1.16x10^6^ cells/ml in serum-free Schneider’s medium. Each dsRNA (0.6 µg/5 µl/well) was loaded onto the empty plates before 30µl of the clone pools were uniformly dispensed into the wells using a multichannel pipette. The plates were incubated for 45 min at 25°Cand tilted in between twice. Thereafter 60µl complete medium was added to each well. The plates were sealed and incubated for 4 days. At day 4 the culture medium was replaced by100μl lysis-buffer and tilted at room temperature for 15 min. 50µl of the lysate was transferred to a new 96-well. The luciferase activities in the cells were measured in the 96-well microplate luminometer (OrionL, Berthold) by injecting 50µl of luciferin into each well. 6-well-plates: The cell pellet of clone pools used for the screen validation was resuspended at 1.5x10^6^ cells/ml in serum-free Schneider’s medium. 1 ml of the clone pools were uniformly dispensed into the wells and 5 µg of dsRNA/well was added to the cells, in case of a double knockdown 5 µg of each dsRNA was added. The plates were incubated for 45 min at 25°C and tilted in between twice. Thereafter 2 ml complete medium was added to each well. The plates were sealed and incubated for 4 days. At day 4 cells were harvested and transferred one half of each for the luciferase assay and the Westernblot analysis. After spinning at 2400 g for 5 min twice, including a washing step, the pellet was resuspended in 250μl lysis-buffer and tilted at room temperature for 15 min. 50µl of the lysate was transferred to a new 96-well plate. The luciferase activity was measured in the 96-well microplate luminometer (OrionL, Berthold) by injecting 50µl luciferin into each well.

*RNAi on other insulation sites*

The experiments were done in 6-well plates (see RNAi hit validation). The stable clone pools with different CTCF binding sites were seeded at 5x10^5^ cells/ml in serum-free Schneider’s medium.

*ChIP*

4x10^7^ S2 cells were fixed in 1% formaldehyde for 15 min at 18°C. The reaction was stopped by adding glycine to a final concentration of 125 mM. After washing, cells were resuspended in SDS lysis buffer (1% SDS, 10 mM EDTA, 50 mMTris-HCl (pH 8.1), protease inhibitors) and incubated 10 min on ice. Chromatin was prepared by shearing with Bioruptor (Diagenode) at setting “high” for 20 cycles with 30 sec “On” and 30 sec “Off” to yield chromatin with a size ranging from 200-800 bp. The sonicated chromatin was diluted 10 fold in dilution buffer (0.01% SDS, 1.1% Triton X-100, 1.2 mM EDTA, 16.7 mMTris-HCl (pH 8.1), 167 mMNaCl, protease inhibitors).

Chromatin was immunoprecipitated by incubating with the appropriate antibodies overnight at 4°C. To collect the antibody/chromatin complexes the solution is incubated for 1 h at 4°C with 30 µl Protein G Plus/Protein A Agarose suspension (Millipore). After washing one time with Low Salt Buffer (0.1% SDS, 1% Triton X-100, 2 mM EDTA, 20 mMTris-HCl (pH 8.1), 150 mMNaCl), one time with High Salt buffer (0.1% SDS, 1% Triton X-100, 2 mM EDTA, 20 mMTris-HCl (pH 8.1), 500 mMNaCl), one time with LiCl Buffer (0.25 M LiCl, 1% NP40, 1% deoxycholate, 1 mM EDTA, 10 mMTris-HCl (pH 8.1)) and two times with TE Buffer (10 mMTris-HCl, 1 mM EDTA (pH 8.0)) the crosslinks were reversed and DNA is recovered by Illustra GFX columns (GE Healthcare). Real-time PCR was performed by using the Rotor-Gene 3000 (Corbett Research) and C1000 Thermal Cycler + CFX96 (Bio Rad). Standard error of the mean was calculated from the average of two PCR repeats for each primer set from three to four biological replicates of each ChIP.

*MNase assay*

4x10^7^ S2 cells were fixed with 0.3% formaldehyde for 3 min at 18°C with shaking and the reaction terminated by addition of 125 mM glycine. The cell pellet is washed first with PBS and second with 1 ml Nuclei Buffer (10 mMTris (pH 7.4), 10 mMKCl, 1.5 mM MgCl_2_, 1 mM DTT, protease inhibitors) and centrifuged after every step at 720 g for 5 min at 4°C. The pellet is resuspended in 1 ml Nuclei Buffer, incubated for 10 min at 4°C and cells disrupted with 20 strokes of a loose pestle. Nuclei were collected by centrifugation as described above and resuspended in 1 ml MNase Buffer (15 mMTris (pH 7.4), 60 mMKCl, 15 mMNaCl, 1 mM CaCl2, 250 mM sucrose, 0.5 mM DTT,). 200 µl aliquots were treated with 250 U MNase (Thermo Scientific #EN0181) for 40 min at 18°C and afterwards the reaction is stopped with 12.5 mM EDTA and 0.5% SDS. Proteins and RNA were degraded, the resulting DNA purified and electrophoresed on an agarose gel. Mononucleosome bands were excised from gel and processed for sequencing.

*Mass Spectrometry*

After immunoprecipitation with a FLAG antibody proteins were eluted from the beads and loaded onto a SDS-PAGE (4-12% Bis-Tris Invitrogen). Gels were stained with colloidal blue (Invitrogen), and evenly sized gel pieces were excised and processed for mass spectrometry. The gel pieces were subjected to in gel reduction and alkylation, followed by trypsin digestion as described previously [[1](#_ENREF_1)]. Briefly, gel pieces were washed twice with 50% (50 mM NH_4_HCO_3_ eluent additive for LC-MS (Sigma-Aldrich) / 50% ethanol) for 20 min and dehydrated with 100% ethanol for 10 min and then vacuum centrifuged. Gel pieces were reduced with 10 mM DTT for 45 min at 56°C and alkylated with 55 mM iodoacetamide for 30 min at RT in the dark. After two steps of washing / dehydration, samples were dehydrated twice with 100% ethanol for 15 min and vacuum centrifuged. Gel pieces were digested overnight at 37°C in 50 µl of digestion buffer containing 12.5 ng/µl of Sequencing Grade Modified Trypsin (Promega Corp., Madison, USA). Released peptides were extracted (collecting separately the liquid mixture of each samples at each step) once by adding 100 µl of 30% acetonitrile LC/MS grade (Thermo Scientific) / 3% trifluoracetic acid (TFA, protein sequence analysis grade, Sigma-Aldrich) in water (LC/MS grade quality, Thermo Scientific), twice by adding 70% acetonitrile, followed by two final extractions with 100% acetonitrile. Extracts were vacuum centrifuged to remove acetonitrile and subsequently acidified with 0.5% TFA. Samples containing tryptic peptides were desalted and concentrated with homemade "STAGE" tips (Stop and Go extraction tips) filled with C-18 (C18 Empore Disks, 3M, Minneapolis, MN) as described earlier [[2](#_ENREF_2), [3](#_ENREF_3)].

Mass spectrometric experiments were performed on a nano-flow HPLC system (Agilent) connected to a LTQ-Orbitrap XL instrument (Thermo Fisher Scientific) equipped with a nano electrospray source (Proxeon). The mass spectrometer was operated in the data dependent mode to monitor MS and MS/MS spectra. Survey full-scan MS spectra (from m/z 300–2000) were acquired in the Orbitrap with a resolution of R=60,000 at m/z 400 after accumulation of 1,000,000 ions. The five most intense ions from the preview survey scan delivered by the Orbitrap were sequenced by collision-induced dissociation (CID) in the LTQ. Mass spectra were analyzed using MaxQuant software (Version 1.1.14.10) [[4](#_ENREF_4)] and all tandem mass spectra were searched against the Drosophila International Protein Index protein sequence database (IPI version r5.1) and concatenated with reversed copies of all sequences. The required false positive rate was set to 1% at the protein and peptide level. Maximum allowed mass deviation was set to 7 ppm in MS mode and 0.5 Da for MS/MS peaks. Cysteine carbamido-methylation was searched as a fixed modification and N-acetyl methionine, and phospho (STY) was searched as variable modifications. A maximum of three missed cleavages were allowed. Protein quantitation was performed with the MaxQuant label free option as described in [[5](#_ENREF_5)].

*Processing of ChIP-seq and MNase-seq reads:*

ChIP-seq and MNase-seq reads were aligned to a pre-compiled dm3 reference index with BOWTIE [[6](#_ENREF_6)] downloaded from the BOWTIE homepage (http://bowtie-bio.sourceforge.net/index.shtml). Unambiguously mapped and unique reads were kept for subsequent analyses. All downstream analyses were done in R/BioConductor (<http://www.bioconductor.org>). We made use of the following R/BioConductor packages: *GenomicRanges* [[7](#_ENREF_7)], *GenomicFeatures* [[8](#_ENREF_8)] and *chipseq* [[9](#_ENREF_9)]. After read extension continuous coverage vectors were calculated for visualization in genome browsers. In order to determine the effect of RNAi treatments on H3 density and MNase protection the dm3 genome was binned into 100 bp bins. For each condition the total number of overlapping reads was determined. Next we used DESeq [[10](#_ENREF_10)] in order to correct for differential library sizes and to calculate fold enrichment/depletion per bin between two conditions (e.g. ISWI RNAi versus luciferase RNAi). After log2-transformation we used these enrichment-vectors in order to calculate average enrichment across certain genomic features (transcriptional start sites, insulator binding sites etc.) in 10 or 20 kb windows.

*Processing of ChIP-chip data (ModEncode):*

Public ChIP-chip data specific for *Drosophila melanogaster* S2 cells was downloaded from ModEncode (http://www.modencode.org/). Data was downloaded in wiggle format containing normalized log2-transformed fold enrichments of specific ChIP over input control. For data related to DREAM complex [[11](#_ENREF_11)] we downloaded CEL-files from NCBI's gene expression omnibus (GSE9087) and processed them using TiMAT (http://bdtnp.lbl.gov/Fly-Net/chipchip.jsp?w=timat) in order to calculate normalized log2-transformed fold enrichments of specific ChIP over input control.

In order to determine the relationship between CP190 binding profiles and other factors we binned the dm3 genome into 100 bp sized bins and calculated for each binding profile the average enrichment for each bin. These enrichment vectors were used to calculate pair wise correlation coefficients. The coefficients were clustered using hierarchical clustering and the observed relationships were plotted as heat maps.

Alternatively above enrichment vectors were used to extract enrichment profiles across 8 kb windows around CP190 binding sites as downloaded from ModEncode (ModEncode ID: 280). Site-specific enrichment profiles for individual factors were concatenated and the resulting vectors were used to partition all CP190 binding sites into sub-groups using k-means clustering. Most informative separation was observed for setting the number of clusters to 6. Binding profiles were re-ordered according to the clustering result and plotted as heat maps.

**Primer sequences**

Oligonucleotide sequences used to generate fragments for *in vitro* transcription were as follows:

(23bp T7 sequence was always to be added)

dCTCF 3'UTR 5'TAATACGACTCACTATAGGGAGATCCTGAAAACAAAAGCTAATGGA3' and 5'TAATACGACTCACTATAGGGAGAAGTGTCGGGGATCATTTACAA3';

eGFP

5'TAATACGACTCACTATAGGGAGAATGGTGAGCAAGGGCGAGGA3'and

5'TAATACGACTCACTATAGGGAGACTTGTACAGCTCGTCCATGC3';

NURF301(DRSC34908)

5'TAATACGACTCACTATAGGGAGACGAGCACTGATACTGCCAAA3'and

5'TAATACGACTCACTATAGGGAGATACCTCCTTAATGATGCGGC3';

ISWI (HFA07446)

5'TAATACGACTCACTATAGGGAGACTCCGGAAAGATGGCTATTC3'and

5'TAATACGACTCACTATAGGGAGAATTAAGCATTTCATCCTTGTTCA3';

DREF (DRSC03319)

5'TAATACGACTCACTATAGGGAGAGGTGTGCAAAAGGTCCTCTT3'and

5'TAATACGACTCACTATAGGGAGAGGGCGTGTCATCAAGCAC3';

Pzg(DRSC11848)

5'TAATACGACTCACTATAGGGAGAAGTGCGGCACTTGTTCGAT3'and

5'TAATACGACTCACTATAGGGAGATTTGCTTCCCGGTTTGGG3';

NURF-38 (HFA04629)

5'TAATACGACTCACTATAGGGAGATCAACGGTGGCACGAAG3'and

5'TAATACGACTCACTATAGGGAGATAGCTTGAAGACCCCTATGA3';

CAF1/p55(BKN23026)

5'TAATACGACTCACTATAGGGAGAGCTTGGCATCTGCTACATGA3'and

5'TAATACGACTCACTATAGGGAGACGCTAATCTTGGCAGTGTGA3';

CAF1/p55 (DRSC30995)

5'TAATACGACTCACTATAGGGAGAGGCAGTCGTCTCGACAATCT3'and

5'TAATACGACTCACTATAGGGAGACCACCATTTCGAGGCTTTTA3';

Mip130(DRSC18575)

5'TAATACGACTCACTATAGGGAGATCACTGCCAGATTAAGATCTCC3'and

5'TAATACGACTCACTATAGGGAGAAGTAGCCGCGATTTCTCGT3';

CAF1-180 (DRSC17807)

5'TAATACGACTCACTATAGGGAGATCGTCCTTGATCTGAAATGG3'and

5'TAATACGACTCACTATAGGGAGAGTCGCCTCAAGCAACAGG3';

Mip40 (BKN24355)

5'TAATACGACTCACTATAGGGAGATTTGGGACAGGATTTCTTCG3'and

5'TAATACGACTCACTATAGGGAGAGATTCGGCATTCGAAATTGT3';

E2F2(BKN26480)

5'TAATACGACTCACTATAGGGAGATGTCCATGCTAACGGGTGTA3'and

5'TAATACGACTCACTATAGGGAGACGCACGAAGAATAGAGGGAG3';

Mip120 (DRSC29182)

5'TAATACGACTCACTATAGGGAGACCTAGACGACACGGAACCAT3'and

5'TAATACGACTCACTATAGGGAGATCACGCCCTTAGAAAGCACT3'

CP190 (MRC014_E10)

5'TAATACGACTCACTATAGGGAGATGCCGGGGACGATGATGACGATGAT3'and

5'TAATACGACTCACTATAGGGAGACTCTTCTGGGCTGCGGCTGTATTTG3'

Luciferase

5'TAATACGACTCACTATAGGGAGAACGATTTTGTGCCAGAGTCC3'and

5'TAATACGACTCACTATAGGGAGAGCCCATATCCTTGCCTGATA3'

**References**

1. Shevchenko A, Tomas H, Havlis J, Olsen JV, Mann M: **In-gel digestion for mass spectrometric characterization of proteins and proteomes.** *Nature protocols* 2006, **1:**2856-2860.

2. Kruger M, Moser M, Ussar S, Thievessen I, Luber CA, Forner F, Schmidt S, Zanivan S, Fassler R, Mann M: **SILAC mouse for quantitative proteomics uncovers kindlin-3 as an essential factor for red blood cell function.** *Cell* 2008, **134:**353-364.

3. Rappsilber J, Friesen WJ, Paushkin S, Dreyfuss G, Mann M: **Detection of arginine dimethylated peptides by parallel precursor ion scanning mass spectrometry in positive ion mode.** *Anal Chem* 2003, **75:**3107-3114.

4. Cox J, Mann M: **MaxQuant enables high peptide identification rates, individualized p.p.b.-range mass accuracies and proteome-wide protein quantification.** *Nature biotechnology* 2008, **26:**1367-1372.

5. Luber CA, Cox J, Lauterbach H, Fancke B, Selbach M, Tschopp J, Akira S, Wiegand M, Hochrein H, O'Keeffe M, Mann M: **Quantitative proteomics reveals subset-specific viral recognition in dendritic cells.** *Immunity* 2010, **32:**279-289.

6. Langmead B: **Aligning short sequencing reads with Bowtie.** *Curr Protoc Bioinformatics* 2010, **Chapter 11:**Unit 11 17.

7. Aboyoun P, Pages H, Lawrence M: **GenomicRanges: Representation and manipulation of genomic intervals.** *(R package version 1125)*.

8. Carlson M, Pages H, Aboyoun P, Falcon S, Morgan M, Sarkar D, Lawrence M: **GenomicFeatures: Tools for making and manipulating transcript centric annotations.** *(R package version 1123 )*.

9. Sarkar D, Gentleman R, Lawrence M, Yao Z: **chipseq: chipseq: A package for analyzing chipseq data.** *(R package version 1101 )*.

10. Anders S, Huber W: **Differential expression analysis for sequence count data.** *Genome Biol* 2010, **11:**R106.

11. Georlette D, Ahn S, MacAlpine DM, Cheung E, Lewis PW, Beall EL, Bell SP, Speed T, Manak JR, Botchan MR: **Genomic profiling and expression studies reveal both positive and negative activities for the Drosophila Myb MuvB/dREAM complex in proliferating cells.** *Genes & Development* 2007, **21:**2880-2896.
